# NADPH oxidase 1 supports proliferation of colon cancer cells by modulating reactive oxygen species-dependent signal transduction

**DOI:** 10.1074/jbc.M116.768283

**Published:** 2017-03-22

**Authors:** Agnes Juhasz, Susan Markel, Shikha Gaur, Han Liu, Jiamo Lu, Guojian Jiang, Xiwei Wu, Smitha Antony, Yongzhong Wu, Giovanni Melillo, Jennifer L. Meitzler, Diana C. Haines, Donna Butcher, Krishnendu Roy, James H. Doroshow

**Affiliations:** From the ‡ Developmental Therapeutics Branch of the Center for Cancer Research,; the ¶ Division of Cancer Treatment and Diagnosis, NCI, National Institutes of Health, Bethesda, Maryland 20892,; the § Department of Medical Oncology and Therapeutics Research and; the ‖ Bioinformatics Group, City of Hope Comprehensive Cancer Center, Duarte, California 91010,; the ** Developmental Therapeutics Program, SAIC-Frederick, Inc., NCI at Frederick, Frederick, Maryland 21702, and; the ‡‡ Pathology/Histotechnology Laboratory, Leidos, Inc./Frederick National Laboratory for Cancer Research, NCI, Frederick, Maryland 21702

**Keywords:** angiogenesis, cell cycle, colon cancer, cyclin D1, mitogen-activated protein kinase (MAPK), NADPH oxidase, protein phosphatase, reactive oxygen species (ROS)

## Abstract

Reactive oxygen species (ROS) play a critical role in cell signaling and proliferation. NADPH oxidase 1 (NOX1), a membrane-bound flavin dehydrogenase that generates O_2_^˙̄^, is highly expressed in colon cancer. To investigate the role that NOX1 plays in colon cancer growth, we used shRNA to decrease NOX1 expression stably in HT-29 human colon cancer cells. The 80–90% decrease in NOX1 expression achieved by RNAi produced a significant decline in ROS production and a G_1_/S block that translated into a 2–3-fold increase in tumor cell doubling time without increased apoptosis. The block at the G_1_/S checkpoint was associated with a significant decrease in cyclin D_1_ expression and profound inhibition of mitogen-activated protein kinase (MAPK) signaling. Decreased steady-state MAPK phosphorylation occurred concomitant with a significant increase in protein phosphatase activity for two colon cancer cell lines in which NOX1 expression was knocked down by RNAi. Diminished NOX1 expression also contributed to decreased growth, blood vessel density, and VEGF and hypoxia-inducible factor 1α (HIF-1α) expression in HT-29 xenografts initiated from NOX1 knockdown cells. Microarray analysis, supplemented by real-time PCR and Western blotting, revealed that the expression of critical regulators of cell proliferation and angiogenesis, including c-MYC, c-MYB, and VEGF, were down-regulated in association with a decline in hypoxic HIF-1α protein expression downstream of silenced NOX1 in both colon cancer cell lines and xenografts. These studies suggest a role for NOX1 in maintaining the proliferative phenotype of some colon cancers and the potential of NOX1 as a therapeutic target in this disease.

## Introduction

Intracellular oxidation-reduction balance in epithelial cells has for more than 30 years been viewed predominantly as a dynamic equilibrium between the production of potentially toxic reactive oxygen species (ROS)^3^ and the detoxification of reactive species by a broad range of antioxidant enzymes and small molecules (1). A more physiological appreciation of the role of ROS has developed over the past decade; it is now clear that low levels of ROS, especially H_2_O_2_, play a critical role in signal transduction for most receptor tyrosine kinases and many cytokines (2, 3). In playing such a role, oxidants may provide potent trophic, rather than antiproliferative, signals required for epithelial cell, including tumor cell growth (4, 5) and angiogenesis (6–8). Recent data are also consistent with the hypothesis that the proliferation of certain epithelial tumors is favored by an elevated oxidant set point, which promotes genetic instability and provides the basis for the inhibition of tumor growth by free radical scavengers (5, 9–11).

The production of ROS by tumor cells *in vitro* and *in vivo* was demonstrated over 20 years ago; at that time, a potential role for tumor cell-related reactive oxygen formation in metastasis, invasion, and the development of tumor cell heterogeneity was postulated (12, 13). However, a comprehensive understanding of the mechanism(s) underlying the formation of reactive oxygen in tumors remained incomplete until the discovery of a family of epithelial NADPH oxidases that are, to varying degrees, structural homologs of gp91*^phox^*, the major membrane-bound component of the respiratory burst oxidase of leukocytes (14). Six members of the NADPH oxidase family of membrane flavin dehydrogenases have been described in addition to gp91*^phox^* (NOX2), the catalytic subunit of the phagocyte oxidase that produces ROS during the process of cellular host defense (15). The biological functions of the *NOX* gene family members, particularly in human cancer, remain incompletely understood (16, 17).

NOX1, originally discovered utilizing Caco2 human colon cancer cells (18), is expressed in both normal and malignant colonic tissue and at lower levels in vascular smooth muscle, uterus, prostate, and osteoclasts (19). The NOX1 catalytic subunit contains binding sites for FAD and NADPH; the N-terminal portion of the molecule contains six hydrophobic segments that form transmembrane α-helices (20). NOX1 associates with membrane-bound p22*^phox^* and soluble subunit analogs of both p47*^phox^* and p67*^phox^* known, respectively, as NOX1 organizer (NOXO1) and NOX1 activator (NOXA1), as well as the small GTPase Rac1, to transfer electrons from intracellular reducing equivalents across the cell membrane, producing O_2_^˙̄^ (21–23). Expression of NOX1, in concert with NOXO1 and NOXA1, in oxidase-deficient cells dramatically increases ROS generation (21).

Evidence linking NOX1 to cytokine-related reactive oxygen production and inflammation provides a critical perspective from which to interpret recent studies of the role of NOX1 in colorectal malignancies (24–26). NOX1 is expressed in relative abundance in the distal colon (27). In patients with ulcerative colitis, who are at increased risk of developing colon cancer (28), the expression of NOX1 is significantly enhanced in the presence of active inflammation (29). Furthermore, NOX1 expression in colonic adenocarcinomas is also significantly higher than in adjacent normal colonic epithelium in a substantial proportion of patients (30, 31).

Current studies suggest that NOX1 plays critical roles in both intestinal host defense (27, 32) and regulation of colonic cell growth and apoptosis, including angiogenesis and malignant transformation (7, 33–38). The presence of NOX1 in surface mucosal cells of the distal large bowel provides an appropriate physiological milieu from which to influence the killing of pathogenic bacteria and the innate immune response (32). In contrast, based on the available experimental evidence, NOX1 also plays an essential role in oxidant-mediated signal transduction involving the RAS/MAPK pathway (34, 35). Furthermore, activated NOX1 in colonic epithelial cells, producing ROS, could contribute to genetic instability (11).

In a previous study, transient knockdown of NOX1 expression with siRNA was shown to produce a modest effect on cell proliferation in HT-29 cells and evidence of enhanced apoptosis in Caco2 human colon cancer cells *in vitro* (39). To clarify the role of NOX1 in colon cancer growth further, we utilized NOX1 shRNA in HT-29 human colon carcinoma cells to evaluate the effect of stable, silenced NOX1 expression on reactive oxygen production, tumor cell proliferation, cell cycle regulation, gene expression, signal transduction, and angiogenesis in both a cell culture model and in HT-29 xenografts. Our results demonstrate that down-regulation of NOX1 expression significantly diminishes reactive oxygen metabolism and markedly decreases the proliferation of HT-29 cells both *in vitro* and *in vivo*. Growth delay of tumor cells following knockdown of NOX1 expression appears to be due to a profound block at the G_1_/S transition of the cell cycle that is related to cyclin D_1_ down-regulation, and alterations in other components of the G_1_ checkpoint that may reflect a protein phosphatase-mediated inactivation of the mitogen-activated protein kinase (MAPK) pathway. Stable NOX1 knockdown is also accompanied by significant alterations in the expression of a broad portfolio of genes critical to cell proliferation and to the angiogenic process. The effect of decreased NOX1 expression *in vivo* is demonstrable both as a significant alteration in the growth of HT-29 xenografts as well as the development of their supporting blood vessels.

## Results

### Down-regulation of NOX1 gene expression decreases reactive oxygen production and tumor cell growth rate in stable clones of HT-29 cells

HT-29 cells were utilized for these experiments because previous investigations had demonstrated that this cell line expressed high levels of NOX1 mRNA (27). We found that the mean mRNA expression ratios of NOX1 or its accessory genes in HT-29 cells (normalized to β-actin) were ≈15,000 (×10^−6^) for NOX1; ≈20,000 (×10^−6^) for NOXO1; and 1100 (×10^−6^) for NOXA1 (Table 1); these experiments, in conjunction with our previous study (40), strongly suggest that among human tumor cells lines, NOX1 is most frequently expressed in those originating from the colon (31). The expression ratios of other members of the NADPH oxidase gene family (NOX2–5, Duox1, and Duox2) in HT-29 cells were either undetectable or <1000 (×10^−6^) (Table 1).

**Table 1:** **Expression of NADPH oxidase isoforms in HT-29 cells and stable clones transfected with shRNAs normalized to β-actin (×10^−6^)** The mRNA expression levels of genes in the NADPH oxidase family were measured by real-time RT-PCR in HT-29 parental cells and in clonal variants expressing a scrambled shRNA sequence (SC) or NOX1 shRNA (6A or G6) during logarithmic growth in cell culture; the method is described under “Experimental procedures.” The data represent the means of triplicate determinations that varied by <15%.

Genes	HT-29	SC	6A	G6
*NOX1*	14,813	17,328	992	4172
*NOXA1*	1113	1241	2514	3116
*NOXO1*	20,402	8914	64,401	43,250
*NOX2*	966	1482	1120	7091
*NOX3*	0	0	3	12
*NOX4*	0	0	0	0
*NOX5*	3	7	183	0
*DUOX1*	78	37	56	9
*DUOX2*	1	5	1	2
*DUOXA2*	0	3	4	6

**Figure 1.**
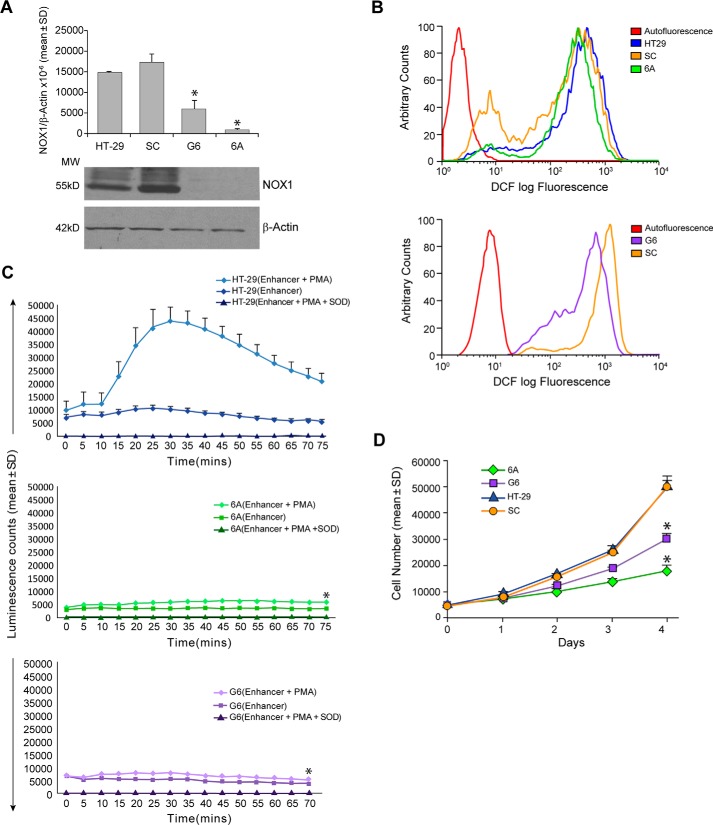
**Down-regulation of NOX1 in HT-29 cells by stable transfection of shRNAs decreases reactive oxygen production and increases tumor cell doubling time.** *A*, *upper panel* demonstrates by real-time RT-PCR the effect of NOX1 shRNA on NOX1 expression in stable clones (6A and G6), as well as the effect of a scrambled target sequence (SC) shRNA on NOX1 expression. NOX1 mRNA levels in parental HT-29 cells are also provided. NOX1 expression is displayed as the mean ± S.D. of the ratio of NOX1/β-actin × 10^−6^ from 3 (for clone G6) or 10 (for clone 6A) separate experiments using cells from passage numbers 9 to 17. *, *p* < 0.001 *versus* either HT-29 cells or cells from the scrambled target sequence clone. The *lower panel* shows the expression of NOX1 protein by Western analysis in HT-29 cells and stable shRNA clones relative to β-actin. *B*, *top panel*, demonstrates intracellular ROS production as detected by analytical cytometry using the redox-sensitive dye CM-H_2_-DCF-DA. A left shift in fluorescence intensity indicates decreasing amounts of ROS; in this panel, steady-state ROS levels are shown for parental HT-29 cells, the SC clone expressing a scrambled target sequence, and clone 6A that expresses a NOX1 shRNA. The *bottom panel* compares intracellular ROS levels in the scrambled SC to that in the G6 NOX1 knockdown cells. *C, upper panel* demonstrates the production of ROS detected as chemiluminescence in the presence or absence of phorbol myristate acetate and superoxide dismutase for parental HT-29 cells over a 75-min time course at room temperature. *Middle panel*, the effect of PMA on ROS formation in the 6A NOX1 knockdown cells was evaluated under identical experimental conditions. *Bottom panel*, G6 NOX1 knockdown cells were evaluated in the same fashion. *Error bars* represent standard deviations from triplicate samples; *, *p* < 0.01 *versus* ROS production from PMA-treated parental HT-29 cells. *D*, tumor cell proliferation was measured by doubling time and by daily cell counts; 5 × 10^5^ cells were plated in duplicate for each cloned line and for parental HT-29 cells; for doubling time measurements, cells were harvested following 72 h of cell culture and counted; cell proliferation was also assessed on a daily basis as shown. The data are expressed as the mean ± S.D. of three independent experiments. *, *p* < 0.01 compared with either HT-29 cells or to cells transfected with the scrambled target sequence.

To down-regulate NOX1 expression, NOX1-specific or scrambled shRNA constructs were developed, transfected into HT-29 cells, and selected in G418. Clones exhibiting stable growth were expanded from cells transfected with target sequence 6 (clones shNOX1 6A and shNOX1 G6) and the scrambled sequence (clone shSC). NOX1 mRNA expression did not change through 20 passages for clones 6A, G6, and SC; levels of NOX1 expression were significantly lower in clones 6A and G6 (Fig. 1*A*) than in parental HT-29 cells or cells expanded from the SC clone (*p* < 0.001 for either 6A or G6 compared with either parental HT-29 cells or cells from the SC clone). NOX1 knockdown resulted in a nearly complete inhibition of NOX1 expression at the protein level (Fig. 1*A*). Furthermore, the decrease in NOX1 expression was not accompanied by significant changes in the expression of any other members of the *NOX* gene family (Table 1) or a variety of important antioxidant genes (catalase, glutathione peroxidase 1, superoxide dismutase, or peroxiredoxin; data not shown).

The oxidative conversion of the non-fluorescent indicator dye 2′,7′-dichlorodihydrofluorescein diacetate (DCF-DA) to the highly fluorescent species 2′,7′-dichlorofluorescein (DCF) was used to measure constitutive levels of ROS in HT-29, SC, 6A, and G6 cells (Fig. 1*B*). DCF production from DCF-DA represents the net effect of cellular oxidant metabolism from all sources, including the mitochondrial electron transport chain and cytoplasmic flavin dehydrogenases, in addition to the NOX1 complex. Inhibition of NOX1 expression in both 6A and G6 cells was associated with a substantial decrease in steady-state ROS levels when compared with the parental HT-29 line or tumor cells derived from the SC clone (Fig. 1*B*). Furthermore, activation of NOX1 activity with PMA (previously shown to enhance ROS production in HEK293 cells with a fully reconstituted NOX1 complex (41)) demonstrated that PMA-enhanced reactive oxygen formation in HT-29 cell clones with significantly diminished NOX1 expression was decreased by ≥80% (Fig. 1*C*), *p* < 0.01. Down-regulation of NOX1 in HT-29 cells was also accompanied by a significant decline in cell proliferation (Fig. 1*D*). The doubling times of G6 and 6A cells, compared with cells from the parental HT-29 line or from clone SC, were increased ≈2–3.5-fold, respectively.

### Inhibition of NOX1 expression produces a G_1_ block in cell cycle progression associated with a decrease in cyclin D_1_ levels that is not accompanied by increased apoptosis

**Figure 2.**
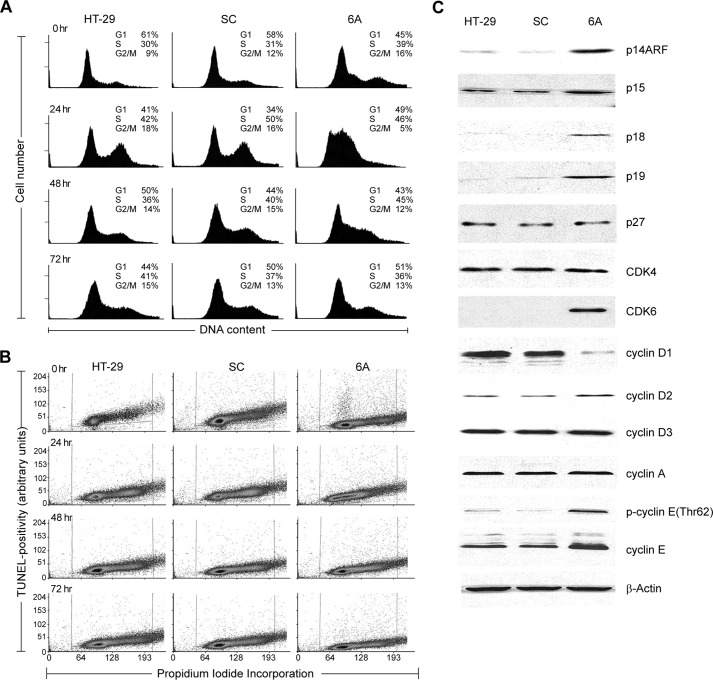
**Suppression of NOX1 expression by shRNA in HT-29 cells alters cell cycle progression and expression of cell cycle regulatory proteins without enhancing apoptosis.** *A*, cell cycle progression was analyzed in HT-29 human colon cancer cells stably transfected with an shRNA against NOX1 (6A) or non-genome specific shRNA (*SC*). HT-29 parental cells are shown as an additional control. Synchronized and exponentially growing cells were analyzed by analytical cytometry after 0, 24, 48, and 72 h. Numerical data correspond to the percentage of cells at the indicated stages of the cell cycle. Data shown are representative of three independent experiments. *B*, analysis of apoptosis and cell cycle progression immediately prior to and after serum stimulation in synchronized HT-29 parental cells and SC and 6A clones. The extent of propidium iodide incorporation (for cell cycle determination) is shown along the *x* axis; the degree of TUNEL positivity throughout the cell cycle is quantitated as events on an arbitrary scale along the *y* axis. *C*, protein levels of cyclins D_1–3_, A and E, Cdk4 and -6, as well as p14, p15, p18, p19, and p27 were examined by Western analysis in logarithmically growing HT-29, SC, and 6A cells. Uniformity of protein loading was confirmed by β-actin expression. Data are representative of 3–5 independent experiments.

To elucidate potential mechanisms responsible for the prolongation of tumor cell doubling time in the 6A cell line, we examined cell cycle progression by flow cytometry (Fig. 2*A*). Parental HT-29 and stable cloned cell lines were synchronized by serum starvation and then either collected or serum-stimulated to re-enter the cell cycle. Cell cycle progression was followed for 72 h; 24 h following release from serum starvation, cells from clone 6A demonstrated a block at the G_1_/S interface, with 5% of the cells progressing into G_2_/M compared with 16–18% of SC or parental HT-29 cells. Consistent with our doubling time results, the parental and SC cells completed a full cell cycle approximately every 24–30 h, whereas 6A cells required 72 h to complete the cycle (Fig. 2*A*). The delay in cell cycle progression for 6A cells was not, however, associated with a substantial increase in apoptosis (Fig. 2*B*). Although there appeared to be a modest increase in TUNEL positivity for 6A cells in G_1_ at the zero time point, the observed G_1_/S block was not associated either with an increase in low molecular weight DNA (sub G_0_/G_1_ DNA) or enhanced TUNEL positivity throughout the cell cycle during the 72 h of observation following stimulation with serum (Fig. 2*B*).

To understand the altered cell cycle progression observed in 6A cells, selected cell cycle proteins were analyzed in stable clones and parental cells during logarithmic cell growth (Fig. 2*C*). The block at the G_1_/S transition is consistent with the observed decrease in cyclin D_1_ protein expression and the increase in expression of the CDK4/6 complex inhibitors p14, p15, p18, and p19 in cells with decreased NOX1 expression. As shown in Table 2, expression levels of p18 and p19 were also increased at the mRNA level.

**Table 2:** **Expression of cell cycle genes in HT-29 cells and stable clones transfected with shRNAs normalized to 18S rRNA (×10^−8^)** The mRNA expression levels of genes related to cell cycle progression were measured by real time RT-PCR in HT-29 parental cells and in clonal variants expressing a scrambled shRNA sequence (SC) or Nox1 shRNA (6A) during logarithmic growth in cell culture; the method is described under “Experimental procedures.” The data represent the means of triplicate determinations that varied by <15%; ND means not determined.

Gene	HT-29	SC	6A
*CDKN1A* (*p21*)	2122	1686	529
*CDKN1B* (*p27*)	2244	1851	2852
*CDKN1C* (*p57*)	264	175	55
*CDKN2A* (*p16*)	35,745	57,868	104,531
*CDKN2B* (*p15*)	561	243	508
*CDKN2C* (*p18*)	83	138	458
*CDKN2D* (*p19*)	518	495	1141
*CDK2*	1637	1814	2158
*CDK4*	7645	7709	8911
*CDK6*	4339	3491	10,959
*SKP2*	2323	4916	7149
*E2F6*	591	577	534
*E2F1*	3993	3661	5475
*RB*	2292	2197	3181
*PCNA*	12,893	16,621	21,015
*RAD51*	2306	2081	2406
*SMAD3*	3558	2652	3720
*SMAD7*	1569	1008	2100
*TGF*β*1*	2056	4731	10,009
*CCNA1* (cyclin A_1_)	ND	ND	ND
*CCNA2*	987	2228	2343
*CCNB1* (cyclin B_1_)	1505	3382	3125
*CCNB2*	15,144	21,218	18,550
*CCNB3*	8	4	5
*CCND1* (cyclin D_1_)	11,711	10,348	8172
*CCND2*	2	1	0
*CCND3*	2557	2625	4795
*CCNE1* (cyclin E_1_)	1796	1412	4272
*CCNE2*	1057	991	2086
*CDC2*	2610	2732	3454
*CHK1*	2287	1984	2321
*CHK2*	710	620	753
*ATM*	228	145	171
*CDC25A*	372	606	640

### Decreased signaling through the Ras/Raf pathway is associated with diminished expression of cyclin D_1_ in NOX1 knockdown colon cancer cells

**Figure 3.**
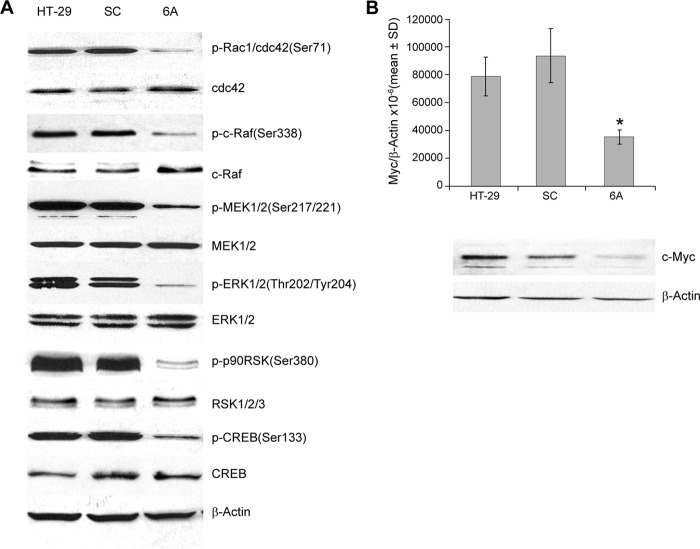
**Effect of NOX1 knockdown on the expression of components of the MAPK pathway and c-Myc.** *A*, components of the Ras/Raf signaling pathway were evaluated by Western analysis in logarithmically growing HT-29 cells and clones from HT-29 cells following selection for a scrambled shRNA sequence (*SC*) or NOX1 knockdown (*6A*); results are representative of 3–5 independent experiments. *B,* effect of silencing NOX1 on the expression of c-Myc in HT-29 cells. *Upper panel,* demonstrates that 6A cells, in which NOX1 expression has been decreased by >80–90%, have significantly diminished c-Myc expression, *p* < 0.05 for three independent experiments. *, *p* < 0.05. The *lower panel* confirms that c-Myc protein levels have also been decreased in the 6A cells, relative to the expression of β-actin.

**Figure 4.**
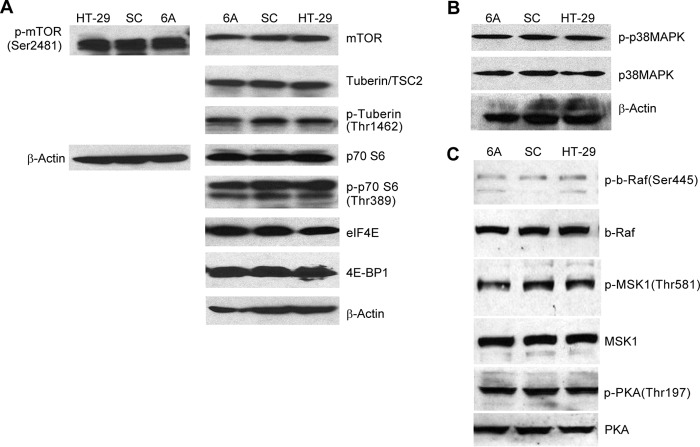
**Effect of NOX1 knockdown on signaling through the Akt and p38 MAPK pathways.** *A*, signaling through the Akt pathway was examined by Western analysis in logarithmically growing HT-29 cells and cells expressing a scrambled shRNA sequence (*SC*) or an shRNA NOX1 knockdown sequence (*6A*); results are representative of three replicate experiments. *B*, effect of NOX1 knockdown on the activation of the p38 MAPK is shown by comparison of 6A cells to cells expressing the scrambled shRNA (*SC*) or the parental HT-29 line. *C*, phosphorylation of other signaling pathways that affect the activation of CREB is shown for 6A, SC, and parental HT-29 cells; the results are illustrative of three independent experiments.

To examine potential mechanisms underlying diminished cyclin D_1_ expression produced by inhibition of NOX1, signal transduction pathways known to control cyclin D_1_ were evaluated in our HT-29 cell clones. When compared with both the parental HT-29 line and control cells transfected with a scrambled shRNA (SC cells), the 6A cell line demonstrated a profound decrease in signaling through the mitogen-activated protein kinase (MAPK) pathway (Fig. 3*A*). Under conditions of logarithmic cell growth in serum, the phosphorylation of essentially all members of this pathway from Rac1 to CREB was markedly decreased. This was associated with a significant decrease in c-Myc mRNA and protein expression (Fig. 3*B*). Under identical experimental conditions, however, NOX1 inhibition produced no significant effect on the Akt and p38 MAPK pathways (Fig. 4, *A* and *B*) nor an effect on alternative signaling pathways of relevance to the activation of CREB (Fig. 4*C*).

### Changes in protein-tyrosine and serine/threonine phosphatase levels help to explain altered MAPK signaling in NOX1 knockdown cells

**Figure 5.**
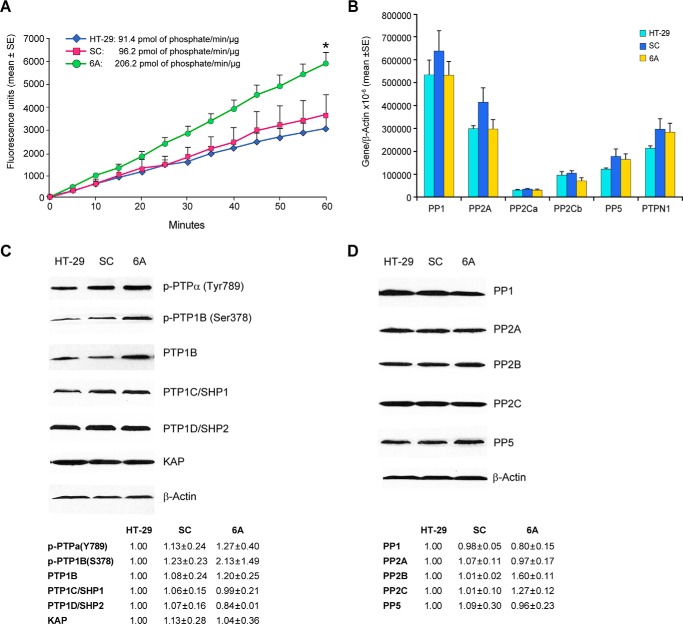
**Effect of NOX1 knockdown on phosphatase activity and expression in HT-29 cells.** *A*, protein-tyrosine phosphatase activity was measured in a kinetic assay as described under “Experimental procedures.” Protein-tyrosine phosphatase activity in the 6A NOX1 knockdown line was significantly higher than in either parental HT-29 cells or cells from the scrambled shRNA clone (*SC*); *, *p* < 0.05 *versus* either SC or parental HT-29 cells. *B*, RNA expression of several serine/threonine protein phosphatases relative to β-actin was determined by real-time RT-PCR in parental HT-29 cells and the SC and 6A clones. *C* and *D,* Western analysis demonstrates the protein expression and phosphorylation status of several members of the protein-tyrosine and serine/threonine phosphatase families, respectively, in HT-29, SC, and 6A cells. As described under “Experimental procedures,” the band intensities have been quantitated; comparisons were made between parental HT-29 cells, the SC scrambled controls, and the 6A knockdown clones. Each intensity calculation is the mean ± S.D. of 2–7 individual experiments.

Because redox-active cysteine moieties play a critical role in the control of protein phosphatase activity (42), we hypothesized that enhanced protein phosphatase activity produced by the decreased ROS levels observed following NOX1 knockdown might help to explain the profound dephosphorylation of the MAPK pathway. We found that for 6A cells total protein-tyrosine phosphatase (PTP) activity levels were doubled, *p* < 0.05 (Table 3 and Fig. 5*A*). Serine/threonine phosphatase activities were increased by ≥50% whether total phosphatase activity levels were measured or those of PP1, PP2A, PP2B, or PP2C. PTP and total serine/threonine phosphatase activity levels were also increased by ≈30% in the shNOX1 G6 clone, *p* < 0.05. The increase in serine/threonine phosphatase activity levels was not associated with a significant change in the mRNA or protein expression of these genes (Fig. 5, *B* and *D*). In like manner, except for a modest, but not significant, increase in PTP1B expression and phosphorylation, we found no change in the protein expression of protein-tyrosine phosphatases in the HT-29 cell clones (Fig. 5*C*).

**Table 3:** **Effect of Nox1 knockdown on protein phosphatase activity in HT-29 cells and stable clones** Protein phosphatase levels in logarithmically growing parental HT-29 cells and in clonal variants expressing a scrambled shRNA sequence (SC) or NOX1 shRNA (6A or G6) were examined for both protein-tyrosine phosphatase and serine/threonine protein phosphatase levels as described under “Experimental procedures.” Experiments were performed at least in triplicate.

Phosphatase	HT-29	SC	6A	G6
Protein-tyrosine phosphatase (total)*^a^*	85.7 ± 1.2	94.7 ± 0.9	185.2 ± 14.8*^b^*	129.7 ± 1.5*^b^*
Serine/threonine phosphatase (total)	100.4 ± 4.4	95.6 ± 2.7	155.4 ± 8.9*^b^*	128.2 ± 6.2*^b^*
PP1	80.8 ± 6.5	86.3 ± 1.4	127.4 ± 2.8*^b^*	
PP2A	74.2 ± 2.8	80.9 ± 1.0	120.1 ± 5.1*^b^*	
PP2B	67.8 ± 0.3	81.7 ± 2.7	125.2 ± 1.7*^b^*	
PP2C	58.1 ± 3.4	76.0 ± 1.9	116.9 ± 1.1*^b^*	

*^a^* Protein phosphatase activity (pmol of phosphate/min/μg) ± S.E. is shown.

*^b^ p* <0.05 is *versus* both HT-29 and shSC control cells.

**Figure 6.**
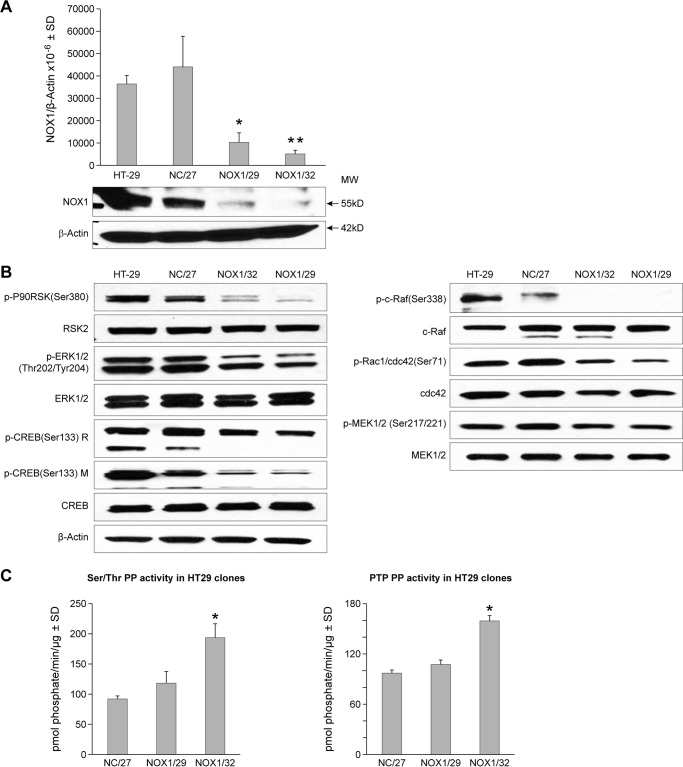
**Effect of NOX1 knockdown using an alternative shNOX1 sequence on the activation of the MAPK pathway and on protein phosphatase activity.** *A*, *upper panel* demonstrates the effect of an alternative NOX1 shRNA on NOX1 expression in stable clones (*NOX1/32* and *NOX1/29*) as well as the effect of a scrambled target sequence (*NC/27*) shRNA on NOX1 expression as measured by quantitative RT-PCR. NOX1 mRNA levels in parental HT-29 cells are also shown. NOX1 expression is displayed as the mean ± S.D. of the ratio of NOX1/β-actin × 10^−6^ for three independent experiments. *, *p* < 0.01 or **, *p* < 0.005 *versus* either HT-29 cells or cells from the scrambled target sequence clone (NC/27). The *lower panel* demonstrates the expression of NOX1 protein by Western analysis in HT-29 cells and stable shRNA clones relative to β-actin; 60 μg of protein were loaded in each lane. *B,* components of the MAPK signaling pathway were evaluated by Western analysis in logarithmically growing HT-29 cells and knockdown or scrambled sequence clones from HT-29 cells; results are representative of three independent experiments. *C*, protein phosphatase activity in logarithmically growing clonal variants of HT-29 cells expressing either a non-targeting sequence (*NC/27*) or shNOX1 (*NOX1/29 and NOX1/32*); *, *p* < 0.001 *versus* NC/27 control cells; results represent three independent experiments.

To examine whether our results with the 6A and G6 NOX1 knockdown clones were specific for NOX1 and unrelated to a non-specific effect of the shRNA used, we silenced NOX1 expression in HT-29 cells using a completely different shRNA silencing vector targeting a different region of the *NOX1* gene; a different scrambled control sequence (not found in the human genome) was also utilized. As shown in Fig. 6*A*, stable 75–90% inhibition of NOX1 expression was demonstrable for shRNA clones NOX1/29 and NOX1/32 when compared with the HT-29 cells stably transfected with the non-targeting sequence (NC/27). When the activation of the MAPK pathway was evaluated in the additional NOX1 knockdown clones (Fig. 6*B*), our findings were very similar to those presented in Fig. 3*A* for 6A cells, namely the activation of MAPK signaling, from phosphorylated c-RAF through activated CREB, was decreased by NOX1 knockdown. Serine/threonine and protein-tyrosine phosphatase activities were also increased in the additional NOX1 knockdown clones, significantly so for NOX1/32 cells (*p* < 0.001).

**Figure 7.**
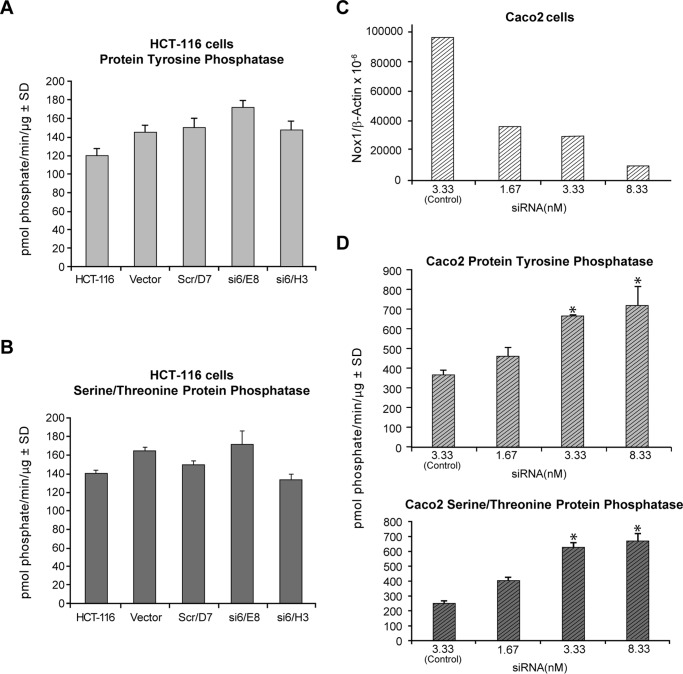
**Effect of NOX1 knockdown on phosphatase activity in HCT-116 and Caco2 colon cancer cells.** *A*, HCT-116 human colon cancer cells were transfected with the identical shRNA vectors employed for experiments with the HT-29 line as outlined under “Experimental procedures”; and vector control clones (*vector*), cells expressing a scrambled shRNA (*Scr/D7*), and two cell lines expressing the NOX1 shRNA (*si6/E8* and *si6/H3*) were examined for protein-tyrosine phosphatase activity levels (*n* = 4). *B*, HCT-116 cells selected for expression of either scrambled or NOX1 shRNAs were studied for the activity levels of serine/threonine protein phosphatase (*n* = 5). *C*, Caco2 human colon cancer cells were transiently transfected with increasing concentrations of siNOX1 constructs (nm) as described under “Experimental procedures.” NOX1 mRNA expression in siRNA-treated cells was determined 24 h later by real-time RT-PCR using β-actin as the reference standard and compared with NOX1 levels in Caco2 cells transfected with a scrambled control siRNA. The data are representative of three independent experiments that varied by <20%. *D*, effect of knocking down NOX1 expression on protein-tyrosine phosphatase levels (*upper panel*) and on serine/threonine protein phosphatase levels (*lower panel*) was examined in triplicate experiments 24 h following transfection with NOX1 siRNA; *, *p* < 0.05 *versus* Caco2 cells transfected with a scrambled control sequence.

To evaluate the specificity of these observations, we studied the effect of stable expression of NOX1 shRNA in HCT-116 cells (that do not express NOX1) on both cell growth and protein phosphatase levels. When stable HCT-116 clones expressing our NOX1 shRNA were compared with the parental line, vector control cells, or cells expressing our scrambled shRNA vector, we could demonstrate no effect of NOX1 shRNA expression on rates of cell growth (data not shown) or on the levels of protein-tyrosine or serine/threonine phosphatase activity in HCT-116 cells (Fig. 7, *A* and *B*). In related experiments, NOX1 expression was decreased by over 80% following transient expression of NOX1 siRNA in Caco2 human colon cancer cells (Fig. 7*C*). We found that both protein-tyrosine and protein-serine/threonine phosphatase activity levels were significantly increased in Caco2 cells following inhibition of NOX1 expression (Fig. 7*D*; *p* < 0.05).

### Microarray, RT-PCR, and Western analyses demonstrate a redox-sensitive gene expression signature that accompanies inhibition of NOX1 expression in stable shRNA-transfected HT-29 clones both in cell culture and in xenografts

**Figure 8.**
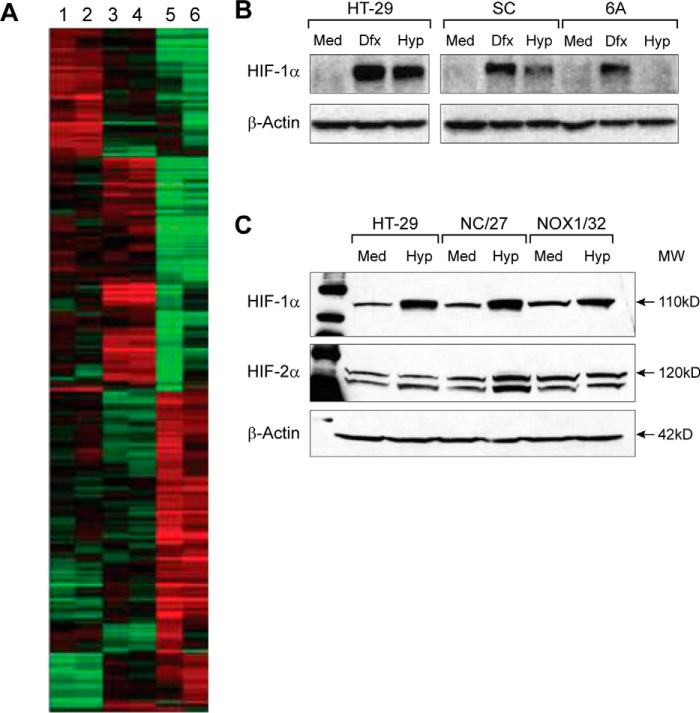
**Evaluation of the effect of NOX1 knockdown on gene expression by microarray analysis in HT-29 cells in tissue culture and in HT-29 xenografts and on HIF-1α and HIF-2α protein expression following exposure to hypoxia.** *A*, normalized gene expression data were clustered using GeneSpring® software. Gene expression levels in parental HT-29, clone SC (scrambled shRNA), and clone 6A cells and xenografts are shown in *green* (down-regulated) and *red* (up-regulated). *Lane 1,* HT-29 parental cells; *lane 2,* clone SC cells; *lane 3,* HT-29 parental xenografts; *lane 4,* clone SC xenografts; *lane 5,* clone 6A cells; and *lane 6,* clone 6A xenografts. Lanes represent the mean expression value of at least three biological replicates. *B*, effect of NOX1 status on the expression of HIF-1α in air (*Med* is exposure to medium only under normoxia for 16 h), or following treatment with deferoxamine (*Dfx* represents 100 μm deferoxamine under normoxic conditions for 16 h), or after hypoxia (*Hyp* is exposure to 1% oxygen for 16 h). HT-29 are parental HT-29 cells; SC are control cells transfected with a scrambled shRNA; and 6A are cells in which NOX1 expression has been decreased by expression of a NOX1 shRNA. Data are representative of three independent experiments. β-Actin was used as the loading control. *C, upper panel* demonstrates the effect of NOX1 knockdown on HIF-1α expression under normoxic and hypoxic conditions evaluated as in *B* with additional HT-29 clones (NC/27 is a NOX1 scrambled clone, and NOX1/32 cells are a NOX1 knockdown line; each was produced using different shRNA targeting sequences than the SC and 6A cells). *C, lower panel* demonstrates the effect of NOX1 knockdown on the expression of HIF-2α in the same cell lines. The data are representative of three independent experiments in which β-actin was used as the loading control; 50 μg of protein were loaded in each lane.

To examine the spectrum of gene expression changes, beyond those associated with altered cell cycle progression, that follows inhibition of NOX1, microarray analysis was performed on HT-29 cells stably transfected with our NOX1 shRNA. We studied both cultured cells and tumor xenografts developed from those cells to determine whether growth conditions modulated the effect of NOX1 inhibition on gene expression. The heat map shown in Fig. 8*A* is a representative experiment from three independent RNA isolations for each cell line and each xenograft; it demonstrates a substantive degree of homogeneity between cell and xenograft gene expression analyses when SC cells are compared with parental HT-29 cells. The expression profile observed for NOX1-silenced 6A cells, whether in tissue culture or when grown as a xenograft, demonstrates major differences when compared with cells stably expressing an irrelevant target sequence. Selected genes for which expression changed at least 2-fold in cells or in xenografts, and which have known effects on tumor cell proliferation, cell cycle regulation, and angiogenesis, or are regulated by ROS, were clustered according to their molecular functions using GeneSpring^TM^ software (supplemental Tables 1 and 2). A complete list of genes from the microarray analysis was deposited in the GEO database with accession number GSE4561. NOX1 silencing was associated with the down-regulation of oncogenes (*MYB* and *MYC*) that play a critical role in cell growth, as well as chemokines (*CCL 14/15* and *CXCR4*) and other genes associated with angiogenesis (*VEGF*, carbonic anhydrase, heme oxygenase). NOX1 down-regulation was also associated with the up-regulation of growth-controlling genes such as *TGF*-β*1*, *AXL* receptor tyrosine kinase (*AXL*) and *CST1* and *CST6*.

To validate the microarray data, real-time PCR analysis was performed for selected genes involved in tumor growth. Expression of the target gene, NOX1, was significantly (*p* < 0.005) inhibited in clone 6A xenografts (26 ± 3; mean ± S.E. of the ratio of the target gene of interest to β-actin × 10^−4^) compared with parental HT-29 cell xenografts (381 ± 28) or SC xenografts (580 ± 48) cells (Table 4). c-*MYC* and c-*MYB* genes, known to play a role in cell cycle regulation and proliferation, were also significantly down-regulated in 6A cells and in 6A xenografts (*p* < 0.05*).* The results for c-MYC were confirmed by Western analysis (Fig. 3*B*). Conversely, expression of TGF-β1, which regulates the expression of two groups of cyclin-dependent kinase inhibitors, INK4 and Cip/Kip, was significantly (*p* < 0.005) up-regulated in 6A cells and xenografts (Table 4).

**Table 4:** **Gene expression in stable clones and xenografts determined by real-time RT-PCR** The mRNA expression levels of genes related to cell proliferation, angiogenesis, and invasion were measured by real-time RT-PCR in HT-29 parental cells and in clonal variants expressing a scrambled shRNA sequence (SC) or Nox1 shRNA (6A) during logarithmic growth in cell culture or from tumor xenografts established in immuno-incompetent mice as described under “Experimental procedures.”

	HT-29	SC	6A	*p* value (HT-29 or SC *vs.* 6A)
**Genes in cell lines**				
**Down-regulated**				
*CCL14/15*	265 ± 79*^a^*	148 ± 48	2 ± 1	<0.05
c-*MYB*	108 ± 7	63 ± 10	13 ± 2	<0.005
c-*MYC*	956 ± 225	758 ± 154	287 ± 75	<0.05
*CXCR4*	5115 ± 1170	2240 ± 87	7 ± 2	<0.005
*FGFR3*	348 ± 53	313 ± 27	15 ± 3	<0.05
*VEGF-A*	402 ± 70	473 ± 79	252 ± 37	<0.05
*HMOX1*	73 ± 25	104 ± 47	30 ± 12	<0.05
*NOX1*	158 ± 20	140 ± 12	13 ± 2	<0.001

**Up-regulated**				
*AXL*	1 ± 0.3	1 ± 0.1	89 ± 11	<0.005
*CDKN2C*	3 ± 1	2 ± 0.3	6 ± 1	<0.05
*CST1*	10 ± 1	10 ± 5	603 ± 157	<0.05
*GAS6*	1 ± 0.3	1 ± 0.1	11 ± 8	<0.05
*TGF-*β*1*	172 ± 24	205 ± 37	623 ± 88	<0.005

**No significant change**				
*ADM*	54 ± 12	26 ± 4	56 ± 10	NS*^b^*
*CAT*	642 ± 31	288 ± 82	534 ± 16	NS
c-*FOS*	540 ± 103	190 ± 20	328 ± 82	NS
*HIF-1*α	109 ± 25	70 ± 14	119 ± 34	NS

**Xenograft genes**				
**Down-regulated**				
*ADM*	151 ± 15	67 ± 9	19 ± 3	<0.005
*CCL14/15*	431 ± 38	424 ± 48	36 ± 13	<0.005
c-*MYB*	47 ± 7	45 ± 13	6 ± 3	<0.05
c-*MYC*	563 ± 48	704 ± 115	246 ± 36	<0.05
c-*FOS*	122 ± 25	103 ± 34	47 ± 18	<0.05
*CXCR4*	40 ± 5	36 ± 5	1 ± 0.2	<0.005
*HMOX1*	43 ± 3	38 ± 4	3 ± 1	<0.005
*NOX1*	381 ± 28	580 ± 48	26 ± 3	<0.005
*VEGF-A*	796 ± 98	751 ± 62	247 ± 35	<0.005

**Up-regulated**				
*AXL*	11 ± 1	4 ± 1	40 ± 6	<0.005
*CDKN2C*	2 ± 0.2	2 ± 0.3	6 ± 2	<0.05
*CST1*	5 ± 1	2 ± 0.3	11 ± 3	<0.05
*GAS6*	3 ± 1	3 ± 0.2	18 ± 3	<0.005
*TGF-*β*1*	303 ± 31	522 ± 26	2124 ± 281	<0.005

**No significant change**				
*CAT*	836 ± 126	494 ± 135	518 ± 78	NS
*FGFR3*	441 ± 37	552 ± 61	331 ± 74	NS
*HIF-1*α	43 ± 7	24 ± 5	16 ± 6	NS

*^a^* Values (mean ± S.E.) represent the ratio of target gene expression to β-actin expression × 10^−4^; data represent a minimum of at least three independent experiments (*n* = 3–15).

*^b^* NS = not significant.

The expression of selected genes that play a critical role in angiogenesis was also examined by real-time PCR. VEGF-A expression was decreased 2-fold in 6A cells when compared with SC or parental cells (Table 4; *p* < 0.05) and was decreased 3-fold in 6A xenografts (*p* < 0.005). Other pro-angiogenic genes, such as adrenomedullin, were also significantly down-regulated in 6A xenografts (Table 4). The expression of heme oxygenase, which is regulated by ROS and VEGF, was also decreased 2–8-fold in cells and xenografts, respectively, in which NOX1 was inhibited (*p* < 0.05). The chemokines CXCR4 and chemokine (C-C motif) ligand 14/15 (CCL14/15), which also play an important angiogenic role, were both significantly down-regulated in cultured cells and in xenografts exhibiting NOX1 silencing (Table 4).

To explore potential mechanisms for the down-regulation of this panel of angiogenic genes, hypoxia-inducible factor 1α (HIF-1α) expression was examined after exposure to hypoxia or to deferoxamine (as a control) in our HT-29 cell lines. As demonstrated in Fig. 8*B*, treatment with the iron chelator deferoxamine enhanced the expression of HIF-1α protein under normoxic conditions in both parental HT-29 cells and the SC scrambled clone. Deferoxamine also increased HIF-1α expression in 6A NOX1 knockdown cells. This might have been expected because, as demonstrated previously in both *Caenorhabditis elegans* (43) and in MDA468 human breast cancer cells (44), deferoxamine functions to limit the availability of iron for the normal function of the proline hydroxylases necessary for the degradation of HIF-1α rather than to diminish the production of H_2_O_2_ (45). However, the induction of HIF-1α by hypoxia was substantively decreased in 6A cells where *NOX1* gene expression is inhibited (Fig. 8*B*).

To confirm these findings, HIF-1α expression under both normoxic and hypoxic conditions was also evaluated in parental HT-29 cells, and in a second NOX1 knockdown clone (NOX1/32) derived from a different shRNA silencing vector, and in an HT-29 clone transfected with a non-targeting sequence (NC/27). As shown in Fig. 8*C* (*top panel*), using a different NOX1 knockdown clone, we also found that the HIF-1α expression was diminished under hypoxic conditions when compared with parental cells or to a scrambled vector control cell line.

In renal cancer cell lines, knockdown of NOX1 expression by siRNA has been found to decrease HIF-2α expression (46) as well as the phosphorylation of Akt and 4E-BP1. Hence, we evaluated the possibility that HIF-2α, as well as HIF-1α, might be affected by NOX1 knockdown. However, Western analysis demonstrated no effect of decreased NOX1 levels on the expression of HIF-2α (Fig. 8*C*, *lower panel*). We also could not show an effect of NOX1 knockdown on the phosphorylation of Akt and 4E-BP1 (data not shown). These results may reflect differences in context between colon and kidney cell lines.

### Down-regulation of NOX1 expression decreases tumor growth, blood vessel development, and the expression of HIF-1α and VEGF in xenografts from HT-29 cell clones

**Figure 9.**
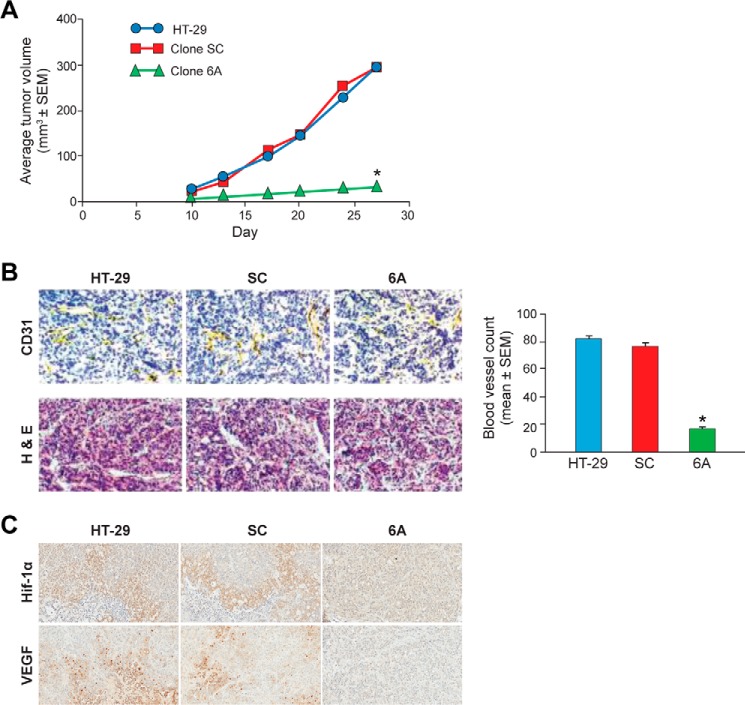
**Decreased NOX1 expression in HT-29 cells diminishes tumor growth, blood vessel formation, and expression of HIF-1α and VEGF in xenografts in athymic mice.** *A*, HT-29 xenografts were established in 6–8-week old male athymic mice (Charles River Laboratories, Frederick, MD). Eight mice in each group were implanted subcutaneously with 0.5 × 10^6^ tumor cells bilaterally. Tumor volumes were calculated from bi-dimensional measurements using the formula 0.5 × length × width^2^. Data points represent mean tumor volumes ± S.E. for 16 tumors per experimental group. Significance was determined using a two-way analysis of variance. The growth rate and tumor volumes in xenografts from 6A cells were significantly decreased compared with control SC cell xenografts or xenografts from the parental HT-29 cell line; *, *p* < 0.01 for either comparison. *B, left panel* demonstrates immunohistochemical analysis of microvessel formation in HT-29 xenografts that was performed on tumors that had been fixed in IHC zinc fixative and stained with CD31 antibody. Representative photomicrographs of the tumors from 6A and SC cells as well as parental HT-29 cells at a magnification of ×200 are shown for xenografts stained with hematoxylin and eosin, as well as for the immunohistochemical evaluation with a CD31 antibody. *Yellowish-brown* peroxidase-positive blood vessels are demonstrated in all xenograft samples. *B*, *right panel* demonstrates the results from three investigators who independently counted the number of blood vessels in five fields per section for eight tumors per condition using a Leica DM IRB microscope at a magnification of ×200. The mean blood vessel count was significantly decreased in xenografts that developed following the implantation of 6A cells; *, *p* < 0.001. *C* demonstrates representative photomicrographs of xenografts evaluated for expression of HIF-1α and VEGF established from parental HT-29 tumor cells, SC scrambled control cells, or 6A NOX1 knockdown cells. Neither HIF-1α nor VEGF expression was quantifiable in any of the seven xenografts established from the 6A NOX1 knockdown cell line.

To study the effect of NOX1 silencing on tumor growth *in vivo*, we established xenografts from parental HT-29 cells and the SC and 6A clonal lines in athymic mice (Fig. 9*A*). Tumors established from 6A cells developed and grew much more slowly than tumors established with SC cells or the parental line. On day 27 after tumor cell implantation, the mean volume of tumor (*n* = 16 in each group; mean ± S.E.) was 34 ± 4 mm^3^ for cells from clone 6A, 305 ± 44 mm^3^ for HT-29 cells, and 298 ± 35 mm^3^ for clone SC cells (*p* < 0.01 for 6A tumor volume *versus* either tumor from SC cells or HT-29 cells). Xenografts were harvested on day 27, and immunohistochemical analyses were performed. The result of studies that employed a CD31 antibody to examine the number of newly formed blood vessels in the tumors are shown in Fig. 9*B*. The mean blood vessel count per field at the time of xenograft collection was significantly higher in tumors that developed from either the HT-29 parental cells (81.9 ± 1.4; mean ± S.E.) or the SC cells (76.3 ± 2.8) than in xenografts arising from 6A cells (16.4 ± 1.5; *p* < 0.001). The diameters of the vessels were also visibly smaller in 6A xenografts than in tumors from either control (Fig. 9*B*). To confirm our RT-PCR studies (Table 4) and to better understand the mechanism underlying the decrease in blood vessel density observed for 6A xenografts, the expression of HIF-1α and VEGF was determined in tumors initiated from parental HT-29, SC, and 6A cells by immunohistochemistry (Fig. 9*C*). On a scale from 0 to 4+ (as described under “Experimental procedures”) HIF-1α expression was 1.88 ± 0.13 (mean ± S.E.; *n* = 8) in parental HT-29 cell xenografts, 1.63 ± 0.20 (*n* = 8) in SC xenografts, and undetectable (0.0 ± 0.0; *n* = 7) in 6A xenografts, *p* < 0.001 *versus* either parental or SA xenografts. In a similar fashion, VEGF expression (as measured by IHC staining intensity and distribution) was 2.13 ± 0.23 (mean ± S.E.; *n* = 8) in the HT-29 parental xenografts, 2.00 ± 0.27 (*n* = 8) in SC xenografts, and undetectable (0.00 ± 0.00; *n* = 7) in the 6A xenografts, *p* < 0.001 *versus* either parental or SC xenografts.

## Discussion

In this study, we have attempted to define the role of NOX1 expression in the control of HT-29 human colon cancer cell growth. We were stimulated to pursue these investigations by the current lack of clarity regarding the role of NOX1 in colon cancer. It has been argued that because NOX1 expression can be enhanced by differentiating agents that decrease tumor cell proliferation, the function of NOX1-induced O_2_^˙̄^ production at the epithelial surface is to enhance host defense rather than mitogenesis (27, 32, 47). However, recent investigations suggest that specific small molecule inhibitors of NOX1 are capable of significantly decreasing the growth of NOX1-containing murine tumors *in vivo* (38). We have recently demonstrated that knock-out of NOX1 prevents the development of chronic colonic inflammation in a genetically engineered mouse model of the pre-malignant condition, inflammatory bowel disease (26). Furthermore, transient knockdown of NOX1 can decrease proliferation of human HT-29 colon cancer cells (39). Finally, NOX1, NOXO1, NOXA1, and p22*^phox^* expression are all significantly increased in colon cancers when compared with simultaneously resected, adjacent, histologically uninvolved colonic epithelium (31). These results support the study of Laurent *et al.* (30) regarding the expression of NOX1 in colon cancer and extend those observations to include the overexpression in colon cancers of the accessory genes (NOXO1, NOXA1, and p22*^phox^*) needed for O_2_^˙̄^ production.

Based on the conclusion that all components of the NOX1 complex are overexpressed in human colon cancer, we focused our current studies on an examination of the role of NOX1 in colon cancer growth in cell culture and *in vivo*. Stable NOX1 knockdown in HT-29 cells produced a significant decrease in baseline- and PMA-stimulated reactive oxygen metabolism (Fig. 1). It is likely that the decrease in ROS levels was due to inhibition of NOX1 and not to an increase in antioxidant defense proteins associated with NOX1 knockdown, because catalase expression was not increased in 6A cells or xenografts compared with controls (Table 4); because the expression of superoxide dismutase, glutathione peroxidase 1, and members of the peroxiredoxin family were also unchanged; and because mRNA levels of the antioxidant enzymes glutathione *S*-transferase (GSTA4) and glutathione peroxidase 3 (GPX3) were 3–4-fold lower in 6A cells *versus* scrambled control cells as assessed by microarray analysis (supplemental Table 1).

In concert with the decrease in intracellular oxidant tone that accompanied shRNA-mediated inhibition of NOX1 expression, we observed a significant decrease in HT-29 growth both *in vitro* and *in vivo*. These results are consistent with previous experiments that have demonstrated increased tumor cell proliferation following NOX1 overexpression (7, 48) and those that have revealed the anti-proliferative effects of decreasing intracellular ROS levels in cell culture and human tumor xenografts with antioxidant proteins or small molecules (5, 49–51). Taken together with our data, these studies support the concept that in some human tumors ROS, rather than being cytotoxic, enhance tumor cell growth and proliferation.

Inhibition of NOX1 expression in HT-29 cells was associated with a marked decrease in cell cycle progression through G_1_ (Fig. 2*A*). Except for a modest increase in TUNEL positivity in serum-starved 6A cells at the G_1_/S interface, the increase in tumor cell doubling time observed following NOX1 inhibition was not accompanied by a substantive degree of apoptosis. Several investigators have suggested that the G_1_ to S transition is a redox-sensitive event (50, 52, 53); however, the precise molecular mechanisms that control the apparent redox sensitivity of the G_1_/S transition remain to be fully elucidated. In our current investigations, we found that the G_1_ block exhibited by 6A cells might be explained by markedly decreased cyclin D_1_ expression, associated with a concomitant up-regulation of members of the INK4 complex that play an inhibitory role in regulating the G_1_ checkpoint (Fig. 2*C*) (54). As demonstrated in Fig. 3*A*, one possible explanation for the decrease in cyclin D_1_ expression is the profound steady-state decrease in MAPK phosphorylation, from c-RAF to CREB (55), that accompanied NOX1 knockdown. CREB plays a critical role in the regulation of cyclin D_1_ expression (56) and is known to be regulated by oxidative stress (57, 58).

Dephosphorylation of the MAPK pathway (including decreased c-RAF activation), which we suggest plays a critical role in slowing the proliferation of HT-29 cells, could be due to enhanced protein-tyrosine and serine/threonine phosphatase activity (Table 3) which may accompany a decrease in intracellular oxidant tone (59, 60). Specific associations between a protein-tyrosine phosphatase (PTP1B) or a serine/threonine phosphatase (PP5) and c-Raf have been described previously (61, 62), albeit not in the context of decreased oxidase gene expression. Further studies, however, will be required to define the underlying mechanism(s) of c-Raf dephosphorylation in our 6A cells.

The specificity of our observations, at least with respect to the effect of NOX1 inhibition on MAPK signaling and protein phosphatase activity, is supported by the development of additional knockdown clones using an shRNA targeting an alternative NOX1 sequence. In these clones, we confirmed that NOX1 knockdown both increased phosphatase activity and decreased MAPK phosphorylation. The lack of effect of NOX1 shRNA expression in the HCT-116 cell line, which does not contain NOX1 mRNA, and our results demonstrating increased phosphatase activity levels in Caco2 cells following transient NOX1 knockdown by siRNA also support a critical role for NOX1 in growth-dependent cell signaling. Finally, in related experiments, we have demonstrated that treatment with low concentrations of the flavin dehydrogenase inhibitor diphenyleneiodonium, which blocks ROS production by NOX1, significantly increased protein phosphatase activity in HT-29 cells but had no effect on the phosphatase activity of the NOX1-deficient HCT-116 line (40).

Transient knockdown of NOX1 expression in Caco2 cells was demonstrated by Wang *et al.* (39) to decrease proliferation by enhancing tumor cell apoptosis rather than by producing a block in cell cycle progression, suggesting that the effects of NOX1 knockdown may vary among tumor cell models. Although this possibility must be acknowledged, it is also possible that the downstream phenotypic consequence of NOX1 knockdown could vary based on the relative expression of pro- or anti-apoptotic genes in human colon cancer cell lines. Caco2 cells are much more susceptible to apoptosis during routine cell culture because they express high levels of pro-apoptotic genes (*BAX, P53*); in contrast, HT-29 cells resist apoptosis, at least in part, because of increased expression of anti-apoptotic genes (*Survivin, MDM2*) (63). In support of this interpretation, we reported that when NOX1 activity is decreased by exposure to diphenyleneiodonium in another NOX1-expressing human colon cancer cell line (LS-174), cell cycle blockade at the G_1_ interface was observed in association with a significant decrease in the expression of cyclins D_1_ and A, results that are very similar to our observations with HT-29 NOX1 knockdown cells (64).

When 6A cells were grown as xenografts in athymic mice, tumors progressed at a significantly slower rate (Fig. 9*A*). Previous work has demonstrated that inhibition of NOX1 significantly interferes with α2β1-integrin signaling as well as the migration of colon cancer cells on collagen (65, 66). It is thus not surprising that the microarray analyses we performed demonstrated that NOX1 knockdown in both cells and xenografts was associated with the up-regulation of members of the cystatin family of protease inhibitors (such as CST1; Table 4) that could decrease tumor cell invasiveness (67) and down-regulation of the c-MYB proto-oncogene (Table 4), which under constitutive circumstances is responsible for blocking differentiation in colon cancer (68). Studies have also suggested that a decrease in intracellular ROS may enhance the expression of the growth inhibitor TGF-β1 (69); we observed an increase in the expression of TGF-β1 in both 6A cells and xenografts (Table 4).

Of particular relevance to colon cancer growth control is the finding of decreased blood vessel density in the HT-29 xenografts from NOX1 knockdown cells (Fig. 9*B*). This decrease in angiogenesis was associated with a profound reduction in the expression of a broad range of pro-angiogenic genes (Table 4; supplemental Table 1), including *VEGF*, *CXCR4*, *HMOX1*, and carbonic anhydrases 9 and 12 (6, 70–72). The down-regulation of VEGF in our NOX1 knockdown xenografts was confirmed at the protein level by immunohistochemistry (Fig. 9*C*). The regulation of all of these genes is, at least in part, under the control of HIF-1α (72). However, current opinion varies widely regarding the role of ROS in the control of HIF-1α expression both under normoxic and hypoxic conditions (45). The undetectable level of HIF-1α protein in HT-29 xenografts developed from NOX1 knockdown cells (Fig. 9*C*) provides support for a relationship between NOX1 levels and HIF-1α-related pro-angiogenic gene expression *in vivo*.

It is appropriate, however, to point out limitations in our understanding of the role of NOX1 in the control of colon cancer growth. HT-29 cells in culture and in xenografts were significantly slowed as they traversed the cell cycle but were not completely blocked at any cell cycle checkpoint by NOX1 inhibition. This might be due to the residual NOX1 activity present despite RNAi; moreover, it is entirely possible that cell cycle progression despite NOX1 knockdown reflects endogenous ROS production from the mitochondrial electron transport chain or other sites that remain available to support the oxidant requirements of HT-29 cells.

**Figure 10.**
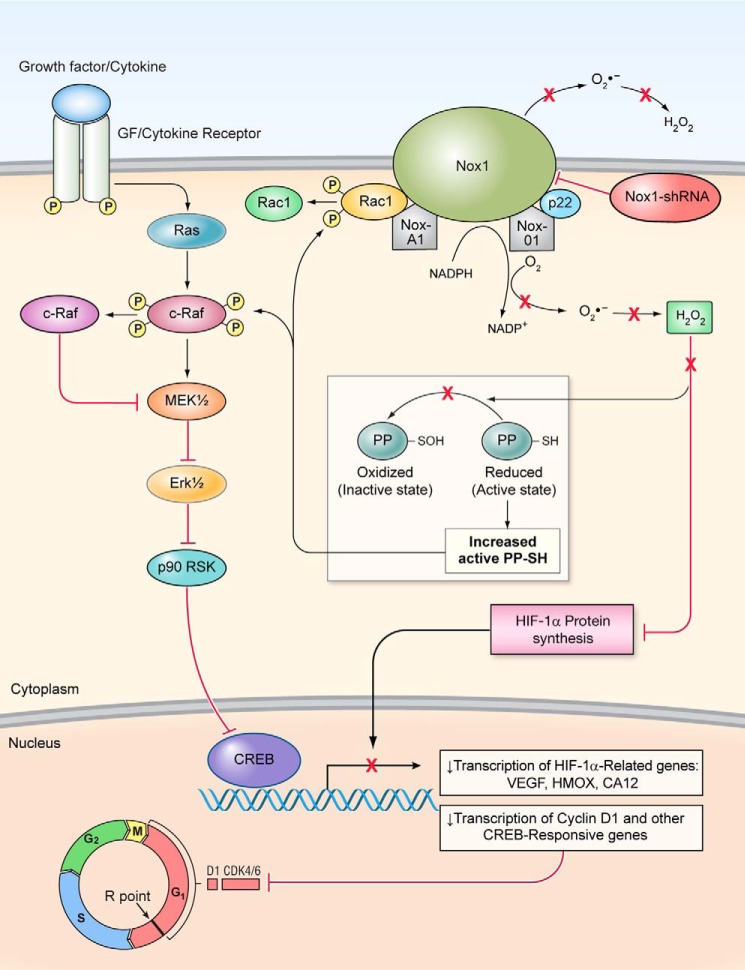
**Model to illustrate how decreased intracellular oxidant tone that accompanies NOX1 inhibition could play a critical role in reducing tumor cell proliferation and angiogenesis in colon cancer cells.** In this model, inhibition of NOX1 expression leads to a decrease in the level of intracellular ROS that favors enhanced protein phosphatase activity. Dephosphorylation of c-Raf by protein phosphatases could deactivate MAPK signaling leading to a cyclin D_1_-related block in cell cycle progression at the G_1_ checkpoint. Concomitantly, diminished intracellular ROS production may alter HIF-1α expression and the transcription of a broad range of HIF-1α-related or ROS-dependent genes critical to the initiation of angiogenesis or tumor cell proliferation. Decreased angiogenesis taken together with diminished cell cycle progression could markedly slow the growth of HT-29 colon cancers *in vivo*.

In summary, as hypothesized in our working model of the role of NOX1-generated ROS in colon cancer growth (Fig. 10), we suggest that some human colon cancers, particularly those with high levels of NOX1 expression, require an oxidative selective pressure to maintain the level of genomic instability required for optimal proliferation, angiogenesis, and/or metastatic potential (9, 11). Interference with ROS homeostasis could, through decreased MAPK signaling, cyclin D_1_ expression, and HIF-1α- or ROS-related gene expression, diminish the growth potential of HT-29 cells. Whatever the ultimate mechanism(s) involved in maintaining a relatively high constitutive reactive oxygen set point, the possibility that some human colon cancers are oxidant-dependent provides a novel perspective from which to develop strategies against this disease, focusing on NOX1 as a therapeutic target.

## Experimental procedures

### Cell culture and RNAi-mediated silencing of the NOX 1 gene

HT-29 human colon cancer cells were purchased from the American Type Culture Collection (Manassas, VA). HT-29 cells were maintained at 37 °C in a humidified atmosphere of 5% CO_2_ in air and were propagated in McCoy's 5A medium with 2 mm l-glutamine (Lonza, Walkersville, MD) and 10% fetal bovine serum (Gemini Bio-Products, West Sacramento, CA). HT-29 cells were transfected with shRNA expression vectors using Lipofectamine 2000 (Life Technologies Inc./Invitrogen) in 1 μg of DNA to 1.5 μl of Lipofectamine 2000 ratio following the manufacturer's recommendations; this resulted in a transfection efficiency of 20–30%. Stable clones of HT-29 cells that had been transfected with vectors containing sequence 6 below or a scrambled target sequence were selected in media containing 700 μg/ml G418 (Sigma). shRNAs were designed against NOX1 (GenBank^TM^ accession number, AF166327; GI, 6138993; mRNA, NM_007052.4) following comparisons with the human genome database (BLAST). Target sequence 6 (948–968 bp), GAGATGTGGGATGATCGTGAC, and a scrambled sequence (not represented in the human genome) GTGTCTTACGTACGGTACGAC were used for these studies. We applied a two-step PCR approach to make the human U6 promoter + siRNA constructs, as described previously (73, 74). The amplified U6 + siRNA cassettes were cloned into the TA-cloning site of pCR2.1 (Life Technologies, Inc./Invitrogen) and then subcloned between KpnI and XbaI restriction sites of pSP73 (Promega Corp., Madison, WI). Finally, the expression cassette was subcloned between the BglII and XhoI restriction sites of a pQBI25-fA1 GFP expression vector (MP Biomedicals/Q-Biogene, Solon, OH). TOP10F′-competent cells (Life Technologies, Inc./Invitrogen) were transformed, and minipreps were sequenced on an ABI 3730 DNA analyzer (Life Technologies/Applied Biosystems, Foster City, CA) using a hot start and dGTP chemistry at the City of Hope Comprehensive Cancer Center DNA Sequencing Core Facility. In addition, NOX1 siRNA sequence-containing plasmids were purchased from Qiagen/SABioSciences (Valencia, CA), catalog no. 336313 KH06068N SureSilencing shRNA vectors, “pGeneClip” with neomycin selection. From the four shNOX1-silencing vectors provided, the following sequence was the most specific: CAAGCTGGTGGCCTATATGAT, located at positions 509–529 nucleotides in the NOX1 gene (NM_007052.4). The non-target sequence, GGAATCTCATTCGATGCATAC, cannot be found in the human genome. The plasmids were prepared according to the manufacturer's instructions. HCT-116 and Caco2 human colon cancer cells were also obtained from ATCC and were propagated as described previously (31). HCT-116 cells were transfected with the NOX1 shRNA (sequence 6) and scrambled sequence shRNA vectors exactly as described for the initial experiments in the HT-29 line; stable clones were also selected in G418. HCT-116 cells were then utilized to examine the effect of NOX1 shRNA expression on cell growth and protein phosphatase levels in a colon cancer line that lacks measurable NOX1 mRNA (40). Caco2 cells express all of the components of the NOX1 protein complex required to produce ROS (31), and several laboratories have confirmed the functional nature of the NADPH oxidase in these cells (27, 75). The Caco2 line was transiently transfected with NOX1 siRNAs to evaluate, in a NOX1-competent cell line in addition to HT-29, the effect of decreasing NOX1 expression on protein phosphatase levels. Caco2 cells were trypsinized, and 1 million cells were either transfected with 3.33 nm scrambled control siRNA or transfected with increasing amounts (1.67–8.33 nm) of siNOX1 (Life Technologies/Ambion, Carlsbad, CA; catalog no. 4392420) in 100 μl of transfection buffer (Amaxa cell line nucleofector kit T) (program B024) utilizing the Amaxa nucleofector device (Lonza, ME).

### RNA isolation and reverse transcription

RNA from cell lines was isolated using an RNeasy mini kit (Qiagen, Valencia, CA). RNA from tissues was isolated after disruption with a Polytron homogenizer using RNA-zol B (Tel-Test Inc., Friendswood, TX) according to the manufacturer's instructions. Genomic DNA contamination was removed with DNase I treatment (Ambion, Austin, TX). Integrity of the RNA was tested on 1% agarose gels (SeaKem, FMC, Rockland, ME) or using Agilent Bioanalyzer 2100 (Agilent Inc., Palo Alto, CA). The RNA concentration and *A*_260_/*A*_280_ ratio were determined by UV spectrophotometry. cDNA was prepared from 1 to 3 μg of RNA using Moloney murine leukemia virus reverse transcriptase enzyme and random hexamers as primers, according to the manufacturer's instructions (Life Technologies, Inc./Invitrogen), and stored at −20 °C until used.

### Real-time RT-PCR

Real-time PCR was carried out to measure the level of gene expression in the colon cancer cell lines. Two μl of cDNA from each sample was used per reaction in a final volume of 20 μl. Primers and probes were designed according to Applied Biosystems guidelines (Primer Express software, Applied Biosystems, Foster City, CA) for the following genes: NADPH oxidase1 (*NOX1*); Nox1 organizer (*NOXO1*); Nox1 activator (*NOXA1*); p22*^phox^*, NADPH oxidase 2–5 (*NOX2–5*); dual oxidases 1 and 2 (*DUOX1* or *2*); adrenomedullin (*ADM*); AXL receptor tyrosine kinase (*AXL*); catalase (*CAT*); chemokine (C-C motif) ligand 15 (*CCL15*); cyclin-dependent kinase inhibitor 2C or p18 (*CDKN2C*); c-*FOS*, c-*MYB*, c-*MYC*, cystatin SN (*CST1*); chemokine (C*X*C motif) receptor 4 (*CXCR4*); fibroblast growth factor receptor 3 (*FGFR3*); growth arrest-specific 6 (*GAS6*); hypoxia-inducible factor 1α subunit (*HIF-1*α); heme oxygenase (decycling) 1 (*HMOX1*); transforming growth factor β1 (*TGF*-β*1*); vascular endothelial growth factor A (*VEGF-A*); and epithelial growth factor receptor (*EGFR*). Genomic DNA amplification was excluded by designing the primers around the exon-intron splicing sites. Other primers and probes were purchased from Applied Biosystems (TaqMan Gene Expression Assays; Applied Biosystems, Foster City, CA) for the following genes: *NOX1* (Hs00246589_m1); β-actin (Hs99999903_m1); *NOXA1* (Hs00611456_g1); *NOXO1* (Hs00376039_g1); p22*^Phox^* (Hs00164370_m1); *RAC1* (Hs00251654_m1); *RAC2* (Hs00427439_g1); *CDKN1A* (p21) (Hs00355782_m1); *CDKN1B* (p27) (Hs00153277_m1); *CDKN1C* (p57) (Hs00175938_m1); *CDKN2A* (p16) (Hs00233365_m1); *CDKN2B* (p15) (Hs00793225_m1); *CDKN2C* (p18) (Hs00176227_m1); *CDKN2D* (p19) (Hs00176481_m1); *CDK2* (Hs00608082_m1); *CDK4* (Hs00175935_m1); *CDK6* (Hs00608037_m1); *SKP2* (Hs00180634_m1); *E2F6* (Hs00242501_m1); *E2F1* (Hs00153451_m1); RB (Hs00153108_m1); *PCNA* (Hs00427214_g1); *RAD51* (Hs00153418_m1); *SMAD3* (Hs00706299_s1); *SMAD7* (Hs00178696_m1); *TGF*-β*1* (Hs00171257_m1); *CCNA1* (cyclin A_1_) (Hs00171105_m1); *CCNA2* (Hs00153138_m1); *CCNB1* (cyclin B_1_) (Hs00259126_m1); *CCNB2* (cyclin B_2_) (Hs00270424_m1); *CCNB3* (cyclin B_3_) (Hs00276946_m1); *CCND1* (cyclin D_1_) (Hs00277039_m1); *CCND2* (cyclin D_2_) (Hs00153380_m1); *CCND3* (cyclin D_3_) (Hs00236949_m1); *CCNE1* (cyclin E_1_) (Hs00233356_m1); *CCNE2* (cyclin E2) (Hs00180319_m1); *CDC2* (Hs00364293_m1); *CHK1* (Hs00176236_m1); *CHK2* (Hs00418065_m1); ATM (Hs00175892_m1); *CDC25A* (Hs00153168_m1); *PPA1*(PP1) (Hs00852097_g1); *PPP2R4* (PP2A) (Hs00221684_m1); *PPM1A* (PP2Cα) (Hs00221372_m1); *PPM1B* (PP2Cβ) (Hs00708683_s1); *PPP5C* (PP5) (Hs00196577_m1); and *PTPN1* (Hs00182260_m1). The PCR amplification was performed on a 384-well plate using the default cycling conditions on an HT7900 Fast Real-time PCR System (Life Technologies, Inc./Applied Biosystems, Foster City, CA). Calibration curves of target and housekeeping genes (*18S* or β-actin) were created using serial dilutions of the plasmids (10^7^ to 1 copy range) containing the gene insert. NOX1 plasmids were kindly provided by Drs. B. Banfi and K.-H. Krause (Geneva, Switzerland) and Dr. H. Kikuchi (Sendai, Japan). Relative gene expression was determined as the ratio of the gene of interest to the internal reference gene expression based on the standard curves.

### Development and characterization of a NOX1 monoclonal antibody

A mouse anti-human NOX1 monoclonal antibody (NOX1-Hyb-Clone-22) was developed by Creative Biolabs, Port Jefferson Station, NY, using the following procedure. A fragmented peptide sequence representing the C-terminal 341 amino acids (224–564 amino acid sequence) of predicted molecular mass of 41 kDa of the human NOX1 protein was expressed in BL21(DE3) host *Escherichia coli*. The purified protein was used as the antigen to produce a monoclonal antibody. Several approaches were used to validate this human monoclonal NOX1 antibody. These included the following: (*a*) transient and stable transfection of HA- or Myc- or FLAG-tagged human NOX1 cDNA into HEK293, COS-7, HT-29, and Caco-2 cells, with evaluation of NOX1 expression by Western analysis using antibodies raised against the HA, Myc, or FLAG tag, as well as the new NOX1 monoclonal antibody; (*b*) the use of NOX1-specific siRNA silencing experiments in cell lines that express reasonably high levels of NOX1, such as HT-29 and LS174T colon carcinoma cells (from the American Type Culture Collection). All of these experiments revealed that NOX1 protein and enzymatic activity corresponded very well with the level of NOX1 mRNA expression. Furthermore, in the course of the experiments to validate the NOX1 antibody, it became clear that the antibody did not cross-react with any other members of the NOX family of proteins (NOX2–5 and Duox1 or -2) that we previously demonstrated to be expressed in other human tumor cell lines (31).

### Western analysis

HT-29 cell clones or parental cells were washed with 1× PBS (Lonza, Walkersville, MD) and harvested during logarithmic phase growth. Whole-cell lysates were prepared in 1× RIPA Lysis Buffer (Millipore/Upstate Biotechnology, Temecula, CA) supplemented with 1 tablet of Complete Mini protease inhibitor and 1 tablet of PhosStop phosphatase inhibitor mix, both from Roche Applied Science. After protein quantitation using the BCA^TM^ protein assay (Thermo Fisher Scientific, Rockford, IL), equal amounts (10–50 μg) of proteins were separated on 4–20% TRIS/glycine gels (Life Technologies, Inc./Invitrogen) and blotted onto PVDF or nitrocellulose membranes using the iBlot^TM^ dry blotting system (Life Technologies, Inc./Invitrogen). Membranes were then blocked with 5% nonfat dry milk in TBST (Quality Biologicals, Gaithersburg, MD; TBS, pH 7.5, containing 0.1% Tween 20) and incubated with a 1:500–5000 dilution of a primary antibody overnight at 4 °C. Kaleidoscope Precision Plus Protein Standards (Bio-Rad) were used as molecular weight markers. For cell cycle protein analysis, the following primary antibodies were obtained from Cell Signaling Technologies Inc., (Danvers, MA): p14/ARF (cs2407); p15 (cs4822); p18 (cs2896); p21 (cs2946); CDK4 (cs2906); CDK6 (cs3136); cyclin A (cs4656); cyclin D_1_ (cs2926); cyclin D_2_ (cs3741); cyclin D_3_ (cs2936); p-cyclin E (Thr-62, cs4136); and cyclin E (cs4129). We obtained the following antibodies from other companies: p19 (catalog no. NA-47, Oncogene Research Products, Boston, MA); p27 (catalog no. 610241, BD Transduction Laboratories). CREB pathway analysis was carried out using Cell Signaling antibodies as follows: p-RAC1/CDC42 (Ser-71) (cs2461); CDC42 (cs2466); p-c-RAF (Ser-338) (cs9427); c-RAF (cs9422); p-MEK1/2 (Ser-217/221) (cs9154); MEK1/2 (cs9126); p-ERK1/2 (Thr-202/Tyr-204) (cs9101); ERK1/2 (cs9102); p-P90RSK (Ser-380) (cs9335); RSK1/2/3 (cs9355); p-CREB (Ser-133) (cs9196); CREB (cs9197); and c-MYC (cs5605). For detection of the expression of Ser/Thr protein phosphatase and protein-tyrosine phosphatase families, the following primary antibodies were utilized: PP1 catalog no. 2167-1); PP2A (catalog no. 1512-1); PP2B (catalog no. 2051-1); p-PTP1B (S378) (catalog no. 216-1), p-PTP1B (S50) (catalog no. 2179-1) from Epitomics Inc.; PP2C was from Novus Biologicals (NBP1-04333); p-PTPα (Y789) (cs4481) was obtained from Cell Signaling; and PP5 (BD611020), PTP1B (BD610139), PTP1C/SHP1 (BD610125), PTP1D/SHP2 (BD610621), and KAP (BD610334) were from BD Transduction Laboratories. The membranes were then washed with TBST and incubated with the appropriate horseradish peroxidase-conjugated secondary antibody (mouse, sc-2055 or rabbit, sc-2054, Santa Cruz Biotechnology, Inc., Santa Cruz, CA) in 1:5–10,000 dilutions (Santa Cruz Biotechnology, Inc.) for 1 h at room temperature. Specific antibody binding was detected using a chemiluminescence detection system (GE Healthcare, Little Chalfont Buckinghamshire, UK), according to the manufacturer's recommendations. In Western analyses, β-actin (catalog no. A3853, Sigma) or GAPDH (Cell Signaling, catalog no. cs2118) was used as the loading control. Band intensities were analyzed using myImageAnalysis Software, version 1.1 (Thermo Fisher Scientific). The target protein bands were normalized to β-actin band intensity, and then bands from parental HT-29 cells were used as the denominator to compare with the band intensities of the NOX1 clones.

### Detection of reactive oxygen species by flow cytometry and chemiluminescence

Production of reactive oxygen in colon cancer cells was examined by flow cytometry using the redox-sensitive dye CM-H_2_-DCF-DA as described previously (76). In brief, cells in logarithmic phase growth, usually 2 days after plating, were harvested, washed with PBS, and counted with a Cellometer Auto T4 (Nexcelom BioSciences, Lawrence, MA); one million cells were resuspended in 500 μl of Hanks' balanced salt solution buffer containing 5 μm dye (Life Technologies/Molecular Probes, Eugene, OR; catalog no. C6827) and incubated at 37 °C for 30 min. ROS were measured with a FACSCalibur flow cytometer (BD Biosciences) at excitation/emission wavelengths of 495 and 527 nm, respectively. The data were analyzed with FlowJo® software (Tree Star Inc., Ashland, OR). ROS production following NOX1 activation by PMA was measured using luminol chemiluminescence with a kit (CS1000, Sigma); briefly, 1 × 10^6^ parental HT-29, 6A, or G6 cells were washed in 1 ml of fresh medium and then resuspended in 100 μl of the supplied assay buffer. Reaction components that contained the luminol solution, a chemiluminescence enhancer, and, where indicated, a final concentration of 200 nm PMA and/or a total of 400 units of superoxide dismutase were added to 96-well plates. The reactions were initiated in triplicate by adding the cell suspension. Luminescence was measured every 2 min for between 1 and 2 h at room temperature using a GloMax^TM^ 96 microplate luminometer (Promega, Madison, WI).

### Doubling time and cell growth assays

Cell lines were harvested and counted with a hemocytometer; 5 × 10^5^ cells were plated in T25 flasks in duplicate. After 72 h, cells were harvested and counted again. Using the formula for exponential cell growth *N_t_* = *N*_0_2*^tf^*, where *t* = 72 h; *N_t_* = number of cells at 72 h; *N*_0_ = initial number of cells; and *f* = cell cycles per unit time; doubling time was calculated as *t*/3.3219(log *N_t_* − log *N*_0_). Average doubling time was calculated for the two replicates of each cell line. The SC, G6, and 6A clones and parental HT-29 cells were also plated in logarithmic phase growth in tissue culture dishes and counted daily using a cell counter (Cellometer Auto T4, Nexcelom Bioscience, Lawrence, MA). Experiments were repeated at least three times with similar results.

### Cell cycle analysis and TUNEL assay

HT-29 cells in logarithmic phase growth in T75 flasks were synchronized by thorough washing in PBS, re-plated in serum-free medium, and grown for 24 h in the absence of serum. Following 24 h of serum starvation, zero time cells were harvested with trypsin and counted. For cells to be examined at subsequent times, media were replaced by complete media with serum; tumor cells were then harvested for analysis at 24, 48, and 72 h. At each time point, harvested cells were counted, and 2–5 × 10^6^ cells were suspended in 0.5 ml of complete media. Cells were added dropwise into 5 ml of ice-cold 1% paraformaldehyde, and incubated on ice for 15 min. The cells were then pelleted, washed in cold PBS, re-pelleted, and finally resuspended in 5 ml of cold 70% ethanol and stored for 1–4 days at −20 °C. Biotin-dUTP was incorporated into 3′-end strand breaks to detect apoptotic cells in a terminal deoxynucleotidyltransferase-mediated dUTP nick end-labeling (TUNEL) reaction. Cells were labeled with avidin-FITC for visualization by flow cytometry and with propidium iodide for concomitant cell cycle analysis. Flow cytometry was performed at the City of Hope Cancer Center Cytometry Core Facility on a MoFlo MLS flow cytometer. Data were acquired using dual laser excitation. Scatter signals were acquired with a HeNe laser (Melles Groit, Carlsbad, CA). All fluorescence excitation was performed at 488 nm using an Innova-90 Argon laser (Coherent, Santa Clara, CA) at 500 milliwatts. FITC emission was measured through a 530DF30 filter. Propidium iodide incorporation was measured through a 640EFLP filter. The two fluorescent signals were separated with a 580DRLP and a 630DRLP dichroic filter. All filters were purchased from Omega Optical (Brattleboro, VT). Data were acquired and analyzed with Summit software (DAKO Cytomation).

### Protein phosphatase assays

Examination of the effect of NOX1 knockdown on PTP levels in HT-29 cells and stable clones, as well as HCT-116 and Caco2 cells, was determined using a PTP end-point assay kit (Millipore, Temecula, CA; catalog no. 17-125). Cells were scraped in lysis buffer containing 20 mm imidazole-HCl, 2 mm EDTA, 2 mm EGTA, pH 7.0, with protease inhibitor mixture (Pierce catalog no. 78425), sonicated, and centrifuged at 2000 × *g* for 5 min. The supernatant protein concentration was measured with a BCA protein assay kit (Pierce catalog no. 23227); from 250 to 750 ng was used per assay well. PTP activity was determined according to the manufacturer's instructions. Briefly, in 96-well (half-volume) plates, protein and 200 μm peptide (RRLIEDAEpYAARG) were added in a 25-μl total volume. After incubation for 15 min, the enzyme reaction was terminated with 100 μl of Malachite Green solution; a subsequent 15 min was allowed for color development, and absorbance was measured at 650 nm with a plate reader (SpectraMax M5; Molecular Devices, Sunnyvale, CA). Enzyme activity was calculated from the amount of released phosphate in picomoles of phosphate/min/μg of protein based on a phosphate standard curve. In some experiments, PTP activity was also determined with a kinetic assay (Life Sciences, Carlsbad, CA; catalog no. R-22067). The reaction was carried out in a 100-μl total volume according to the manufacturer's instructions. Reaction plates contained the fluorescent substrate 6,8-difluoro-4-methylumbelliferyl phosphate, which has excitation/emission maxima at 358/452 nm, respectively. After determination of cell lysate protein content, from 500 to 750 ng of protein was added in a 20-μl volume to the substrate, which was dissolved by adding 80 μl buffer (25 mm MOPS, pH 7.0, containing 50 mm NaCl, 1 mm DTT, and 0.05% Tween 20) to each well; fluorescence was measured after a 20-min initial incubation, every 5 min for 1 h. Enzyme activity was calculated from the phosphate standard curve.

Measurement of serine/threonine protein phosphatase levels was also performed with a commercially available assay kit (Millipore, Temecula, CA; catalog no. 17-127). Samples were prepared in imidazole buffer; to measure phosphatase activity, 200 μm peptide (KRpTIRR) was used as the substrate as outlined above. The reaction was carried out using from 250 to 750 ng of protein per well; enzyme activity was determined after 15 min. The reaction was terminated by addition of 100 μl of Malachite Green solution and then measured and calculated as outlined for PTPs. Specific serine/threonine phosphatase activities were determined as follows: PP-1 detection required addition of DTT and MnCl_2_ (2 nm and 200 μm concentrations, respectively). PP2A determinations required NiCl_2_ in a 1 mm final concentration; and PP2B required 10 μg/ml calmodulin in addition to NiCl_2_ for maximum activity. PP2C detection was carried out by adding DTT, EGTA, and MgCl_2_ in final concentrations of 2, 1, and 20 mm, respectively.

### HIF-1α protein expression

The expression of HIF-1α in parental HT-29 cells, as well as SC and 6A cells, was evaluated under normoxic as well as hypoxic (1% oxygen) conditions using an *In vivo*_2_ 400 hypoxic work station (Ruskinn Technologies, Cincinnati, OH). The expression of HIF-1α in parental HT-29 cells, as well as in a second NOX1 knockdown clone (NOX1/32) derived from a different shRNA silencing vector, and in an HT-29 clonal line transfected with a non-targeting sequence (NC/27), was also evaluated under normoxic and hypoxic conditions using a Modular Incubator Chamber (Billups-Rothenberg, Inc., Del Mar, CA). For these experiments, tumor cells in logarithmic phase growth were serum-starved for 16 h; the HT-29, SC, or 6A cells were then exposed to deferoxamine (100 μm) or media alone in air or to 1% oxygen for 16 h in the hypoxic work station. The effect of deferoxamine on HIF-1α expression was not studied for the NC/27 control cells or the NOX1/32 knockdown line. Whole-cell lysates were prepared, and immunoblots were analyzed for HIF-1α expression (HIF-1α monoclonal antibody was from BD Biosciences) as described previously with β-actin as the loading control (36). Using a similar process, the expression of HIF-2α was also evaluated in HT-29 parental cells and in the NC/27 and NOX1/32 lines under normoxic and hypoxic conditions (HIF-2α antibody from Cell Signaling, catalog no. cs7096).

### HT-29 colon cancer xenografts

Human tumor xenografts were established by subcutaneous injection of 0.5 × 10^6^ cells bilaterally into the flanks of 6–8-week old male athymic mice (Charles River Laboratories, Wilmington, MA). Ten days after implantation, as soon as tumors were palpable, bi-dimensional measurements were recorded for 14–17 days, and tumor volumes were calculated using the formula 0.5 × length × width^2^. Tumor measurements were performed for 14–17 days in these experiments because animals carrying tumors from either parental HT-29 cells or cells stably transfected with the scrambled NOX1 shRNA routinely developed early signs of skin ulceration over their xenografts at this time. Thus, at the end of the 14–17-day test period (24–27 days following the injection of tumor cells), mice were euthanized, and tumors were harvested. A portion of each tumor was fixed (see below) and embedded in paraffin. The remainder was preserved in RNAlater RNA stabilization reagent (Qiagen, Valencia, CA) or frozen at −70 °C for subsequent extraction of RNA or protein for gene expression analysis.

### CD31 immunohistochemistry and blood vessel analysis

Tumors were fixed in IHC zinc fixative (Pharmingen), and tissue sections were prepared (5 μm). Blood vessel growth was detected with a CD31 antibody (rat anti-mouse antibody; catalog no. 553371 from Pharmingen; 1:200 dilution). Tissue sections were stained using standard immunoperoxidase procedures (with 3,3′-diaminobenzidine) at the City of Hope Comprehensive Cancer Center Pathology Core Facility. Slides were counterstained with hematoxylin. Slides were initially scanned at low power (×10–100) using a Leica DM IRB microscope. Regions of new blood vessel formation were then examined, and photomicrographs were obtained at ×200 magnification by three investigators blinded as to sample identity. Each investigator separately quantified the number of blood vessels per field (five fields per section) for each of eight tumors per condition. The blood vessel counts for each individual tumor were scored by averaging the counts in the five fields.

### Immunohistochemical analysis of VEGF and HIF-1α expression in HT-29 xenografts

IHC staining for VEGF expression was performed as follows. Slides were deparaffinized and rehydrated to H_2_O through graded ethanol. Endogenous peroxidase was blocked by incubating slides in 0.6% H_2_O_2_ in methanol for 15 min. Antigen retrieval was then performed by microwaving slides in EDTA, pH 8.0, for 10 min at 100 °C, followed by 20 min of cooling. An avidin/biotin blocking kit (Vector Laboratories catalog no. SP-2001) was used to block endogenous biotin, and then normal goat serum was applied to the sections for 20 min. Anti-VEGF (human, catalog no. AbD Serotec catalog no. AAM51, diluted 1:800 in BSA/PBS) was applied for 60 min at room temperature. Slides were subsequently rinsed in PBS, and secondary antibody (biotinylated goat a/rabbit IgG; Vector Laboratories) was applied for 30 min at room temperature. Sections were rinsed with PBS and then incubated with ABC (Elite Standard, Vector Laboratories) for 30 min. Following a PBS rinse, the slides were placed into 3,3′-diaminobenzidine tetrahydrochloride for 5 min, rinsed with tap water, counterstained with hematoxylin, dehydrated, cleared with xylene, and coverslipped.

IHC staining for HIF-1α expression was performed as follows. Slides were deparaffinized and rehydrated to H_2_O through graded ethanol. Endogenous peroxidase was blocked by incubating slides in 0.6% H_2_O_2_ in methanol for 15 min. Antigen retrieval was then performed with MW Citrate Buffer (BioGenex catalog no. HK086-9K) for 10 min at 100 °C, followed by 20 min of cooling. Sections were rinsed with distilled H_2_O and Tween/PBS (T-PBS). 10% normal goat serum in T-PBS was applied to the sections for 60 min. Slides were then incubated with anti-HIF-1α (Abcam catalog no. ab2185, diluted 1:8000 in BSA/PBS) overnight at room temperature. Slides were subsequently rinsed in T-PBS, and secondary antibody (biotinylated goat a/rabbit IgG; Vector Laboratories, diluted 1:200 in 10% normal goat serum/T-PBS) was applied for 30 min at room temperature. Sections were rinsed with T-PBS and then incubated with ABC (Elite Standard, Vector Laboratories) for 30 min. Following a PBS rinse, the slides were placed into 3,3′-diaminobenzidine tetrahydrochloride for 5 min, rinsed with tap water, counterstained with hematoxylin, dehydrated, cleared with xylene, and coverslipped.

Images were scanned into ScanScope CS (Aperio, Buffalo Grove, IL) at ×40. Images were evaluated and graded for staining intensity by a board-certified veterinary pathologist. Scoring of IHC for VEGF was as follows: most VEGF reactivity was cytoplasmic; 0 = absence of immunoreactivity; 1+ represented very few high-powered fields (hpfs) with a positive reaction; 2+ represented some hpfs positive with one or multiple positive cells; 3+ represented the presence of many positive hpfs positive with single or multiple positive cells; 4+ represented presence of positive staining in almost all hpfs with single or multiple positive cells. Scoring of IHC for HIF-1α was as follows: 0 = absence of immunoreactivity; 1+ represented very few hpfs with a positive reaction and some fields had multiple positive nuclei; 2+ represented some hpfs positive and some hpfs had multiple positive nuclei; 3+ represented positive staining in almost all of the hpfs, but not all nuclei per hpf were positive; 4+ represented the presence of positive HIF-1α staining in all hpfs and nuclei.

### Microarray analysis

Samples of total RNA from the parental HT-29 cell line, stably transfected tumor cell line clones SC and 6A, and from xenografts developed from all three cell lines were analyzed on Affymetrix HG:U133A 2.0 arrays at the DNA and Protein MicroArray Facility (University of California, Irvine), following prescribed protocols in the Affymetrix GeneChip® Expression Analysis Technical Manual. The results were quantified and analyzed with GCOS 1.2 software (Affymetrix, Inc.) using default values. Quality assessment, preprocessing, and statistical analyses of gene expression data were performed using the R statistical language and environment and Bioconductor packages. Data analysis was improved using statistical models such as the Robust Multi-Chip Average method. Genes with an expression value of <80 were excluded from further analysis. The Bioconductor package “limma” was used to identify the genes differentially expressed between the 6A and SC samples. The R-contributed libraries limma provided the necessary routines for fitting linear models to microarray data that incorporate appropriate empirical Bayes smoothing of variances and for making multiple testing adjustments using the false discovery rate (FDR). We used a FDR of <0.05 and a fold change of >1.5 to identify differential expression. Genes exceeding these criteria between SC *versus* HT-29 samples were excluded due to possible transfection effects. Hierarchical clustering using average linkage with the Pearson correlation was applied to differentially expressed gene expression data using the clustering features in GeneSpring version 7.2 (Agilent, Palo Alto, CA). The genes showing altered expression were then categorized on the basis of their cellular components, biological processes, molecular functions, and signaling pathways using the Database for Annotation, Visualization, and Integrated Discovery 2.0 (DAVID 2.0, 17), GeneSpring, and Ingenuity Pathways Analysis (Ingenuity, Mountain View, CA).

### Statistical analysis

Two-tailed Student's *t* tests were performed; values of *p* < 0.05 were considered significant; *NS* designates values that were not significant. Xenograft data were analyzed using a two-way ANOVA. All values presented are the mean ± S.E. or S.D. of at least three experiments.

## Author contributions

J. H. D. and A. J. designed the study; J. H. D. with assistance from A. J. wrote the paper; S. M. performed the xenograft studies; S. G. performed critical Western analyses; D. C. H. and D. B. performed immunohistochemical studies; S. G. and H. L. provided essential technical assistance. X. W. provided informatics support. G. M. performed studies related to HIF-1α expression. J. L., G. J., S. A., Y. W., J. L. M., and K. R. provided critical scientific input to the experiments. All authors reviewed the results, provided essential reviews of the manuscript, and approved the final version of the paper.

## Supplementary Material

Supplemental Data

## Footnotes

3 The abbreviations used are:

ROS: reactive oxygen species
PMA: phorbol myristate acetate
FDR: false discovery rate
DCF: 2′,7′-dichlorofluorescein
DCF-DA: 2′,7′-dichlorodihydrofluorescein diacetate
hpf: high-powered field
IHC: immunohistochemistry
PTP: protein-tyrosine phosphatase
SC: scrambled clone
CREB: cAMP-response element-binding protein.
