# Supplementary material for: NADPH oxidase 1 supports proliferation of colon cancer cells by modulating reactive oxygen species-dependent signal transduction
Source: J Biol Chem. 2017 Mar 22;292(19):7866–87. doi: 10.1074/jbc.M116.768283 (PMC5427267; doi:10.1074/jbc.M116.768283)
Supplement: Supplemental Data [file supp_292_19_7866__index.html]

NADPH Oxidase 1 Supports Proliferation of Colon Cancer Cells by Modulating Reactive Oxygen Species-Dependent Signal Transduction — NADPH oxidase 1 supports proliferation of colon cancer cells by modulating reactive oxygen species-dependent signal transduction — NOX1 modulates colon cancer growth and angiogenesis — Supplemental Data 

# NADPH oxidase 1 supports proliferation of colon cancer cells by modulating reactive oxygen species-dependent signal transduction

## Supplemental Data

- Supplemental Table 1 (.docx, 42 KB) - Supplemental Table 1
- Supplemental Table 2 (.docx, 42 KB) - Supplemental Table 2
