# Supplementary material for: NADPH oxidase 1 supports proliferation of colon cancer cells by modulating reactive oxygen species-dependent signal transduction
Source: J Biol Chem. 2017 Mar 22;292(19):7866–87. doi: 10.1074/jbc.M116.768283 (PMC5427267; doi:10.1074/jbc.M116.768283)
Supplement: Supplemental Data [file 10.1074_M116.768283_jbc.M116.768283-1.docx]

**SUPPLEMENTAL TABLE 1**

|  |  |  |  |  |  |  |  |  |  |  |  |  |  |  |  |  |  |
| --- | --- | --- | --- | --- | --- | --- | --- | --- | --- | --- | --- | --- | --- | --- | --- | --- | --- |
| **Down-regulated genes in cells and xenografts sorted by function**  The fold change in gene expression determined by microarray analysis, as described in “Experimental  Procedures,” is shown for the comparison of HT-29 parental, and SC scrambled clones and xenografts,  compared with cells and xenografts from cells stably transfected with a Nox1 shRNA (6A); NS is not significant. | | | | | | | | |  |  |  |  |  |  |  |  |  |
| Gene Symbol | Accession No. | Fold change in cells | Fold change in xenografts | Gene description | | | | |  |  |  |  |  |  |  |  |  |
| ***Cancer*** |  |  |  |  |  |  |  |  |  |  |  |  |  |  |  |  |  |
| FOS | BC004490 | NS | 2.5 | v-fos FBJ murine osteosarcoma viral oncogene homolog | | | | |  |  |  |  |  |  |  |  |  |
| MYB | NM_005375 | 4.6 | 4.3 | v-myb myeloblastosis viral oncogene homolog (avian) | | | | |  |  |  |  |  |  |  |  |  |
| MYC | NM_002467 | 2.0 | 2.1 | v-myc myelocytomatosis viral oncogene homolog (avian) | | | | |  |  |  |  |  |  |  |  |  |
| RAB26 | NM_014353 | NS | 3.3 | RAB26, member RAS oncogene family | | | |  |  |  |  |  |  |  |  |  |  |
| TNS | NM_018274 | 6.0 | 3.8 | tensin | |  |  |  |  |  |  |  |  |  |  |  |  |
| VEGF | AF022375 | 1.7 | 3.6 | vascular endothelial growth factor | | | |  |  |  |  |  |  |  |  |  |  |
| ***Cell cycle*** |  |  |  |  |  |  |  |  |  |  |  |  |  |  |  |  |  |
| CDKN1A | NM_000389 | NS | 2.3 | cyclin-dependent kinase inhibitor 1A (p21, Cip1) | | | | |  |  |  |  |  |  |  |  |  |
| CDKN1C | N33167 | NS | 3.9 | cyclin-dependent kinase inhibitor 1C (p57, Kip2) | | | | |  |  |  |  |  |  |  |  |  |
| ***Immunity*** |  |  |  |  |  |  |  |  |  |  |  |  |  |  |  |  |  |
| AZGP1 | D90427 | 12.8 | 13.9 | alpha-2-glycoprotein 1, zinc | | |  |  |  |  |  |  |  |  |  |  |  |
| CCL14 ///15 | AF031587 | 30.1 | 6.5 | chemokine (C-C motif) ligand 14 /// 15 | | | |  |  |  |  |  |  |  |  |  |  |
| CCL20 | NM_004591 | NS | 4.7 | chemokine (C-C motif) ligand 20 | | |  |  |  |  |  |  |  |  |  |  |  |
| CEACAM5 | NM_004363 | NS | 7.7 | carcinoembryonic antigen-related cell adhesion molecule 5 | | | | | |  |  |  |  |  |  |  |  |
| CEACAM7 | NM_006890 | NS | 6.6 | carcinoembryonic antigen-related cell adhesion molecule 7 | | | | | |  |  |  |  |  |  |  |  |
| F5 | NM_000130 | 8.7 | 1.6 | coagulation factor V (proaccelerin, labile factor) | | | | |  |  |  |  |  |  |  |  |  |
| GPA33 | NM_005814 | NS | 6.4 | glycoprotein A33 (transmembrane) | | | |  |  |  |  |  |  |  |  |  |  |
| TBXAS1 | NM_030984 | 4.1 | 2.9 | thromboxane A synthase 1 | | |  |  |  |  |  |  |  |  |  |  |  |
| ***Enzymes*** |  |  |  |  |  |  |  |  |  |  |  |  |  |  |  |  |  |
| AKR1B10 | NM_020299 | 28.4 | 16.1 | aldo-keto reductase family 1, member B10 | | | | | |  |  |  |  |  |  |  |  |
| AKR1C1 | NM_001353 | 5.1 | 2.3 | aldo-keto reductase family 1, member C1 | | | | | | | | | | |  |  |  |
| ANPEP | NM_001150 | 2.1 | 23.1 | alanyl (membrane) aminopeptidase, CD13, p150) | | | | | | | | | | |  |  |  |
| ASNS | NM_001673 | 2.5 | 4.1 | asparagine synthetase | | |  |  |  |  |  |  |  |  |  |  |  |
| CA12 | NM_001218 | 11.9 | 11.5 | carbonic anhydrase XII | | |  |  |  |  |  |  |  |  |  |  |  |
| CA9 | NM_001216 | NS | 3.8 | carbonic anhydrase IX | | |  |  |  |  |  |  |  |  |  |  |  |
| DDC | NM_000790 | 1.6 | 14.0 | dopa decarboxylase (aromatic L-amino acid decarboxylase) | | | | | |  |  |  |  |  |  |  |  |
| DPP4 | NM_001935 | 2.8 | 8.6 | dipeptidylpeptidase 4 (CD26) | | | | | | |  |  |  |  |  |  |  |
| DUSP5 | U16996 | NS | 3.4 | dual specificity phosphatase 5 | | |  |  |  |  |  |  |  |  |  |  |  |
| GPD1 | NM_005276 | NS | 3.0 | glycerol-3-phosphate dehydrogenase 1 (soluble) | | | | |  |  |  |  |  |  |  |  |  |
| GPX3 | AW149846 | NS | 3.5 | glutathione peroxidase 3 (plasma) | | | |  |  |  |  |  |  |  |  |  |  |
| GSTA4 | NM_001512 | 4.4 | 3.3 | glutathione S-transferase A4 | | |  |  |  |  |  |  |  |  |  |  |  |
| HK2 | AI761561 | NS | 4.1 | hexokinase 2 | |  |  |  |  |  |  |  |  |  |  |  |  |
| HMOX1 | NM_002133 | 1.9 | 1.7 | heme oxygenase (decycling) 1 | | |  |  |  |  |  |  |  |  |  |  |  |
| HSD17B2 | NM_002153 | NS | 12.7 | hydroxysteroid (17-beta) dehydrogenase 2 | | | |  |  |  |  |  |  |  |  |  |  |
| IDH2 | AU151428 | 2.5 | 3.6 | isocitrate dehydrogenase 2 (NADP+), mitochondrial | | | | |  |  |  |  |  |  |  |  |  |
| NOX1 | NM_007052 | 6.2 | 19.2 | NADPH oxidase 1 | |  |  |  |  |  |  |  |  |  |  |  |  |
| PAPSS2 | NM_004670 | 8.3 | 3.2 | 3'-phosphoadenosine 5'-phosphosulfate synthase 2 | | | | |  |  |  |  |  |  |  |  |  |
| SERPINE2 | AL541302 | NS | 20.9 | serine (or cysteine) proteinase inhibitor, clade E2 | | | | | | | | | |  |  |  |  |
| SHMT2 | AW190316 | 2.4 | 3.1 | serine hydroxymethyltransferase 2 (mitochondrial) | | | | |  |  |  |  |  |  |  |  |  |
| SI | NM_001041 | NS | 15.7 | sucrase-isomaltase (alpha-glucosidase) | | | |  |  |  |  |  |  |  |  |  |  |
| SMP3 | NM_025163 | 3.2 | 12.7 | SMP3 mannosyltransferase | | |  |  |  |  |  |  |  |  |  |  |  |
| ST6GAL1 | AI743792 | 3.9 | 3.9 | ST6 beta-galactosamide alpha-2,6-sialyltranferase 1 | | | | |  |  |  |  |  |  |  |  |  |
| ***Nucleic acid binding proteins*** | | |  |  |  |  |  |  |  |  |  |  |  |  |  |  |  |
| ADM | NM_001124 | NS | 7.0 | adrenomedullin | |  |  |  |  |  |  |  |  |  |  |  |  |
| BGN | AA845258 | 5.9 | 2.8 | biglycan /// serologically defined colon cancer antigen 33 | | | | |  |  |  |  |  |  |  |  |  |
| BHLHB2 | NM_003670 | NS | 3.0 | basic helix-loop-helix domain containing, class B, 2 | | | | |  |  |  |  |  |  |  |  |  |
| CREB3L2 | BE675139 | 3.2 | 1.8 | cAMP responsive element binding protein 3-like 2 | | | | |  |  |  |  |  |  |  |  |  |
| FOXA2 | AB028021 | NS | 5.3 | forkhead box A2 | |  |  |  |  |  |  |  |  |  |  |  |  |
| GNAI1 | AL049933 | 4.8 | 2.3 | guanine nucleotide binding protein (G protein), | | | | | | | |  |  |  |  |  |  |
| KCNH2 | AB044806 | 1.9 | 3.2 | potassium voltage-gated channel, subfamily H member 2 | | | | | |  |  |  |  |  |  |  |  |
| LGALS4 | NM_006149 | 10.7 | 2.1 | lectin, galactoside-binding, soluble, 4 (galectin 4) | | | | |  |  |  |  |  |  |  |  |  |
| MGA | BE502432 | 4.8 | 3.9 | MAX gene associated | | |  |  |  |  |  |  |  |  |  |  |  |
| NR1I2 | NM_003889 | NS | 3.7 | nuclear receptor subfamily 1, group I, member 2 | | | | |  |  |  |  |  |  |  |  |  |
| TNRC9 | AK025084 | 48.1 | 3.2 | trinucleotide repeat containing 9 | | |  |  |  |  |  |  |  |  |  |  |  |
| ***Signal transduction*** | |  |  |  |  |  |  |  |  |  |  |  |  |  |  |  |  |
| ANXA10 | AF196478 | 36.4 | NS | annexin A10 | |  |  |  |  |  |  |  |  |  |  |  |  |
| ANXA13 | NM_004306 | NS | 29.6 | annexin A13 | |  |  |  |  |  |  |  |  |  |  |  |  |
| CTGF | M92934 | 3.1 | 4.2 | connective tissue growth factor | | |  |  |  |  |  |  |  |  |  |  |  |
| CXCR4 | L01639 | 12.0 | 45.1 | chemokine (C-X-C motif) receptor 4 | | | |  |  |  |  |  |  |  |  |  |  |
| EFNA1 | NM_004428 | NS | 2.8 | ephrin-A1 |  |  |  |  |  |  |  |  |  |  |  |  |  |
| FGFR3 | NM_000142 | 12.4 | 1.9 | fibroblast growth factor receptor 3 (thanatophoric dwarfism) | | | | | | |  |  |  |  |  |  |  |
| FGFR4 | AF202063 | 2.8 | 1.7 | fibroblast growth factor receptor 4 | | | |  |  |  |  |  |  |  |  |  |  |
| IGFBP2 | NM_000597 | NS | 17.4 | insulin-like growth factor binding protein 2, 36kDa | | | | |  |  |  |  |  |  |  |  |  |
| INSR | AA485908 | NS | 3.2 | Insulin receptor | |  |  |  |  |  |  |  |  |  |  |  |  |
| JAG1 | U77914 | 2.1 | 2.1 | jagged 1 (Alagille syndrome) | | |  |  |  |  |  |  |  |  |  |  |  |
| LGALS2 | NM_006498 | NS | 8.5 | lectin, galactoside-binding, soluble, 2 (galectin 2) | | | | | | | | | |  |  |  |  |
| NDRG1 | NM_006096 | NS | 17.3 | N-myc downstream regulated gene 1 | | | |  |  |  |  |  |  |  |  |  |  |
| ***Others*** |  |  |  |  |  |  |  |  |  |  |  |  |  |  |  |  |  |
| TFPI | AF021834 | 4.7 | 2.7 | tissue factor pathway inhibitor (coagulation inhibitor) | | | | | | |  |  |  |  |  |  |  |
| TPM1 | M19267 | 2.5 | 2.8 | Tropomyosin 1 (alpha) | | |  |  |  |  |  |  |  |  |  |  |  |
| ***Structural*** |  |  |  |  |  |  |  |  |  |  |  |  |  |  |  |  |  |
| AKAP7 | AL137063 | 13.5 | 67.3 | A kinase (PRKA) anchor protein 7 | | | |  |  |  |  |  |  |  |  |  |  |
| CFTR | NM_000492 | NS | 3.2 | cystic fibrosis transmembrane conductance regulator | | | | | | | | |  |  |  |  |  |
| ECM1 | U65932 | NS | 3.3 | extracellular matrix protein 1 | | |  |  |  |  |  |  |  |  |  |  |  |
| ITPR2 | NM_002223 | 2.9 | 5.6 | inositol 1,4,5-triphosphate receptor, type 2 | | | |  |  |  |  |  |  |  |  |  |  |
| SEPP1 | NM_005410 | NS | 14.1 | selenoprotein P, plasma, 1 | | |  |  |  |  |  |  |  |  |  |  |  |
| ***Transport proteins*** | |  |  |  |  |  |  |  |  |  |  |  |  |  |  |  |  |
| ATP2A3 | AA877910 | 2.2 | 3.5 | ATPase, Ca++ transporting, ubiquitous | | | |  |  |  |  |  |  |  |  |  |  |
| ATP7B | NM_000053 | 2.3 | 6.0 | ATPase, Cu++ transporting, beta polypeptide | | | | | |  |  |  |  |  |  |  |  |
| FABP1 | NM_001443 | NS | 15.6 | fatty acid binding protein 1, liver | | |  |  |  |  |  |  |  |  |  |  |  |
| HEPH | NM_014799 | NS | 85.8 | hephaestin, ferroxidase | | |  |  |  |  |  |  |  |  |  |  |  |
| KCTD12 | AI718937 | NS | 17.3 | potassium channel tetramerisation domain containing 12 | | | | |  |  |  |  |  |  |  |  |  |
| SLC11A2 | AF046997 | NS | 3.1 | solute carrier family 11 (H^+^-coupled metal ion transporters)2 | | | | | | |  |  |  |  |  |  |  |
| SLC26A2 | AI025519 | NS | 3.7 | solute carrier family 26 (sulfate transporter), member 2 | | | | |  |  |  |  |  |  |  |  |  |
| SLC27A2 | NM_003645 | 1.7 | 6.9 | solute carrier family 27 (fatty acid transporter), member 2 | | | | |  |  |  |  |  |  |  |  |  |
| SLC3A1 | NM_000341 | NS | 44.5 | solute carrier family 3 (amino acid transporters) 1 | | | | | | | | | | | | | |
| SLC43A1 | NM_003627 | 5.5 | 4.5 | solute carrier family 43, member 1(neutral amino acid) | | | | |  |  |  |  |  |  |  |  |  |
| SLC5A1 | NM_000343 | NS | 8.8 | solute carrier family 5 (sodium/glucose cotransporter) 1 | | | | | |  |  |  |  |  |  |  |  |
| TFRC | NM_003234 | 2.3 | 3.2 | transferrin receptor (p90, CD71) | | |  |  |  |  |  |  |  |  |  |  |  |
| **Unclassified** |  |  |  |  |  |  |  |  |  |  |  |  |  |  |  |  |  |
| ABP1 | NM_001091 | NS | 9.4 | amiloride binding protein 1 (amine oxidase (Cu-containing)) | | | | | |  |  |  |  |  |  |  |  |
| AGT | NM_000029 | 2.8 | 2.4 | angiotensinogen (serine (or cysteine) proteinase inhibitor) | | | | | | | | | | |  |  |  |
| DDIT4 | NM_019058 | NS | 8.4 | DNA-damage-inducible transcript 4 | | | |  |  |  |  |  |  |  |  |  |  |
| ENG | NM_000118 | 2.8 | 1.8 | endoglin (Osler-Rendu-Weber syndrome 1) | | | |  |  |  |  |  |  |  |  |  |  |
| MPRG | NM_017705 | 2.4 | 3.9 | membrane progestin receptor gamma | | | |  |  |  |  |  |  |  |  |  |  |
| SPRY2 | NM_005842 | 2.7 | 3.8 | sprouty homolog 2 (Drosophila) | | |  |  |  |  |  |  |  |  |  |  |  |
