# Supplementary material for: NADPH oxidase 1 supports proliferation of colon cancer cells by modulating reactive oxygen species-dependent signal transduction
Source: J Biol Chem. 2017 Mar 22;292(19):7866–87. doi: 10.1074/jbc.M116.768283 (PMC5427267; doi:10.1074/jbc.M116.768283)
Supplement: Supplemental Data [file 10.1074_M116.768283_jbc.M116.768283-2.docx]

**SUPPLEMENTAL TABLE 2**

| **Up-regulated genes in cells and xenografts sorted by function**  The fold change in gene expression determined by microarray analysis, as described in “Experimental  Procedures,” is shown for the comparison of HT-29 parental, and SC scrambled clones and xenografts,  compared with cells and xenografts from cells stably transfected with a Nox1 shRNA (6A); NS is not significant. | | | | | | | | | |  |  |  |  |  |  |  |
| --- | --- | --- | --- | --- | --- | --- | --- | --- | --- | --- | --- | --- | --- | --- | --- | --- |
| Gene Symbol | Accession No. | Fold change in cells | | Fold change in xenografts | Gene description | |  |  |  |  |  |  |  |  |  |  |
| ***Apoptosis*** |  |  | |  |  |  |  |  |  |  |  |  |  |  |  |  |
| --- | AW474434 | 8.4 | | NS | --- |  |  |  |  |  |  |  |  |  |  |  |
| CASP1 | U13700 | NS | | 2.4 | caspase 1, apoptosis-related cysteine protease | | | | | | | |  |  |  |  |
| CD14 | NM_000591 | 7.3 | | 4.0 | CD14 antigen | | | | |  |  |  |  |  |  |  |
| TNFSF10 | NM_003810 | 8.7 | | NS | tumor necrosis factor (ligand) superfamily, member 10 | | | | | | | | | | | |
| ***Cancer*** |  |  | |  |  |  |  |  |  |  |  |  |  |  |  |  |
| MYBL1 | AW592266 | NS | | 6.5 | v-myb myeloblastosis viral oncogene homolog like 1 | | | | | |  |  |  |  |  |  |
| PIAS3 | NM_006099 | 1.6 | | 3.1 | protein inhibitor of activated STAT3 | | | |  |  |  |  |  |  |  |  |
| RARRES1 | NM_002888 | 7.0 | | NS | retinoic acid receptor responder (tazarotene induced) 1 | | | | |  |  |  |  |  |  |  |
| RARRES3 | NM_004585 | NS | | 4.0 | retinoic acid receptor responder (tazarotene induced) 3 | | | | |  |  |  |  |  |  |  |
| RUNX1 | BF432501 | 2.4 | | 2.3 | runt-related transcription factor 1 (aml1 oncogene) | | | | | | |  |  |  |  |  |
| ***Cell cycle*** |  |  | |  |  |  |  |  |  |  |  |  |  |  |  |  |
| CDKN2C | NM_001262 | 2.2 | | 3.5 | cyclin-dependent kinase inhibitor 2C (p18, inhibits CDK4) | | | | | |  |  |  |  |  |  |
| CDKN2D | U20498 | 1.6 | | 1.7 | cyclin-dependent kinase inhibitor 2D (p19, inhibits CDK4) | | | | | |  |  |  |  |  |  |
| TGFB1 | BC001830 | 4.2 | | 2.6 | transforming growth factor beta 1 induced transcript 1 | | | | |  |  |  |  |  |  |  |
| ***Chaperone*** |  |  | |  |  |  |  |  |  |  |  |  |  |  |  |  |
| ARHGDIB | NM_001175 | 2.4 | | 2.4 | Rho GDP dissociation inhibitor (GDI) beta | | | |  |  |  |  |  |  |  |  |
| CD74 | K01144 | 3.3 | | NS | CD74 antigen ( class II antigen-associated) | | | | | | | | |  |  |  |
| EPB41L1 | AL121895 | 2.6 | | 3.7 | erythrocyte membrane protein band 4.1-like 1 | | | | |  |  |  |  |  |  |  |
| NEDD9 | AL136139 | 4.5 | | 1.7 | neural precursor cell expressed, down-regulated 9 | | | | | |  |  |  |  |  |  |
| SVIL | NM_003174 | 3.6 | | 1.7 | supervillin |  |  |  |  |  |  |  |  |  |  |  |
| ***Enzymes*** |  |  | |  |  |  |  |  |  |  |  |  |  |  |  |  |
| ALDH1A3 | AF198444 | 3.6 | | NS | Aldehyde dehydrogenase 1 family, member A3 | | | | |  |  |  |  |  |  |  |
| ALOX5 | NM_000698 | 4.5 | | 2.1 | arachidonate 5-lipoxygenase | | |  |  |  |  |  |  |  |  |  |
| DUSP10 | N36770 | 3.3 | | 2.2 | dual specificity phosphatase 10 | | |  |  |  |  |  |  |  |  |  |
| HPGD | NM_000860 | 4.7 | | 2.0 | hydroxyprostaglandin dehydrogenase 15-(NAD) | | | | |  |  |  |  |  |  |  |
| INPP4B | NM_003866 | 5.1 | | NS | inositol polyphosphate-4-phosphatase, type II, 105kDa | | | | |  |  |  |  |  |  |  |
| KYNU | D55639 | 3.1 | | 6.6 | kynureninase (L-kynurenine hydrolase) | | | |  |  |  |  |  |  |  |  |
| PLAU | NM_002658 | 2.4 | | 2.6 | plasminogen activator, urokinase | | | |  |  |  |  |  |  |  |  |
| PPP2R3A | AL389975 | 3.4 | | 1.7 | protein phosphatase 2, regulatory subunit B'', alpha | | | | | |  |  |  |  |  |  |
| PTGS2 | NM_000963 | 7.0 | | NS | prostaglandin-endoperoxide synthase 2 (cox-2) | | | | | | | | |  |  |  |
| TGM2 | AL031651 | NS | | 7.2 | transglutaminase 2 (C polypeptide) | | | | | | | |  |  |  |  |
| ***Immunity proteins*** | |  | |  |  |  |  |  |  |  |  |  |  |  |  |  |
| ALCAM | BF242905 | 2.4 | | 7.9 | Activated leukocyte cell adhesion molecule | | | |  |  |  |  |  |  |  |  |
| COCH | AA669336 | 3.5 | | 2.4 | coagulation factor C homolog, cochlin | | | | | |  |  |  |  |  |  |
| IFI44L | NM_006820 | NS | | 3.8 | interferon-induced protein 44-like | | |  |  |  |  |  |  |  |  |  |
| IFI16 | AF208043 | NS | | 5.2 | interferon, gamma-inducible protein 16 | | | |  |  |  |  |  |  |  |  |
| KIAA0992 | NM_016081 | 2.5 | | 4.5 | palladin |  |  |  |  |  |  |  |  |  |  |  |
| ***Nucleic acid binding proteins*** | | | |  |  |  |  |  |  |  |  |  |  |  |  |  |
| ARL7 | BC001051 | | 2.4 | 4.6 | ADP-ribosylation factor-like 7 | | |  |  |  |  |  |  |  |  |  |
| ELK3 | AW575374 | | 1.5 | 2.5 | ELK3, ETS-domain protein (SRF accessory protein 2) | | | | |  |  |  |  |  |  |  |
| FOXC1 | AU145890 | | 3.3 | 2.4 | Forkhead box C1 | |  |  |  |  |  |  |  |  |  |  |
| TRIM29 | NM_012101 | | 3.1 | 1.5 | tripartite motif-containing 29 | | |  |  |  |  |  |  |  |  |  |
| VGLL1 | NM_016267 | | 3.9 | 1.7 | vestigial like 1 (Drosophila) | | |  |  |  |  |  |  |  |  |  |
| ZNF211 | NM_006385 | | 3.1 | 2.1 | zinc finger protein 211 | | |  |  |  |  |  |  |  |  |  |
| ***Signal transduction*** | | |  |  |  |  |  |  |  |  |  |  |  |  |  |  |
| AXL | NM_021913 | | 37.9 | 5.3 | AXL receptor tyrosine kinase | | |  |  |  |  |  |  |  |  |  |
| GAS6 | NM_000820 | | 12.6 | 2.7 | growth arrest-specific 6 | | |  |  |  |  |  |  |  |  |  |
| GPR87 | NM_023915 | | NS | 6.0 | G protein-coupled receptor 87 | | |  |  |  |  |  |  |  |  |  |
| IGFBP6 | NM_002178 | | NS | 3.2 | insulin-like growth factor binding protein 6 | | | |  |  |  |  |  |  |  |  |
| NMU | NM_006681 | | 8.4 | 7.7 | neuromedin U | |  |  |  |  |  |  |  |  |  |  |
| NRP1 | BE620457 | | NS | 5.1 | neuropilin 1 | |  |  |  |  |  |  |  |  |  |  |
| SOSTDC1 | AI927000 | | 7.3 | NS | sclerostin domain containing 1 | | |  |  |  |  |  |  |  |  |  |
| TACSTD2 | J04152 | | 7.5 | NS | tumor-associated calcium signal transducer 2 | | | | |  |  |  |  |  |  |  |
| WNT11 | NM_004626 | | NS | 3.0 | wingless-type MMTV integration site family, 11 | | | | | |  |  |  |  |  |  |
| ***Others*** |  | |  |  |  |  |  |  |  |  |  |  |  |  |  |  |
| CDH11 | D21254 | | NS | 3.5 | cadherin 11, type 2, OB-cadherin (osteoblast) | | | | |  |  |  |  |  |  |  |
| CST1 | NM_001898 | | 29.5 | NS | cystatin SN | |  |  |  |  |  |  |  |  |  |  |
| CST4 | NM_001899 | | 3.2 | NS | cystatin S |  |  |  |  |  |  |  |  |  |  |  |
| CST6 | NM_001323 | | 8.5 | 6.4 | cystatin E/M | |  |  |  |  |  |  |  |  |  |  |
| RGC32 | NM_014059 | | 4.0 | NS | response gene to complement 32 | | | |  |  |  |  |  |  |  |  |
| S100A2 | NM_005978 | | 5.6 | NS | S100 calcium binding protein A2 | | |  |  |  |  |  |  |  |  |  |
| TPBG | NM_006670 | | NS | 3.4 | trophoblast glycoprotein | | |  |  |  |  |  |  |  |  |  |
| ***Structural proteins*** | | |  |  |  |  |  |  |  |  |  |  |  |  |  |  |
| CAV1 | NM_001753 | | 2.9 | 2.3 | caveolin 1, caveolae protein, 22kDa | | | |  |  |  |  |  |  |  |  |
| MMP14 | X83535 | | NS | 2.3 | matrix metalloproteinase 14 (membrane-inserted) | | | | |  |  |  |  |  |  |  |
| MUC16 | NM_024690 | | NS | 7.9 | mucin 16 |  |  |  |  |  |  |  |  |  |  |  |
| ADAM28 | NM_021778 | | 5.0 | 1.7 | a disintegrin and metalloproteinase domain 28 | | | | |  |  |  |  |  |  |  |
| KRT13 | NM_002274 | | 11.2 | 12.3 | keratin 13 |  |  |  |  |  |  |  |  |  |  |  |
| ***Transport proteins*** | | |  |  |  |  |  |  |  |  |  |  |  |  |  |  |
| AP1S2 | NM_003916 | | 3.8 | 1.9 | adaptor-related protein complex 1, sigma 2 subunit | | | | |  |  |  |  |  |  |  |
| CLIC5 | AL049313 | | 3.8 | 1.7 | Chloride intracellular channel 5 | | |  |  |  |  |  |  |  |  |  |
| SLC39A8 | AB040120 | | NS | 3.5 | solute carrier family 39 (zinc transporter), member 8 | | | | |  |  |  |  |  |  |  |
| SLC6A14 | NM_007231 | | NS | 12.0 | solute carrier family 6 (neurotransmitter transporter),14 | | | | | |  |  |  |  |  |  |
| SLCO1B3 | NM_019844 | | NS | 9.3 | solute carrier organic anion transporter family, 1B3 | | | | | |  |  |  |  |  |  |
| TCN1 | NM_001062 | | 12.1 | 3.8 | transcobalamin I (vitamin B12 binding protein) | | | | | |  |  |  |  |  |  |
| TRPC1 | NM_003304 | | NS | 3.7 | transient receptor potential cation channel, C, 1 | | | | | |  |  |  |  |  |  |
| ***Unclassified*** |  | |  |  |  |  |  |  |  |  |  |  |  |  |  |  |
| C10orf116 | BC004471 | | 4.1 | 2.8 | chromosome 10 open reading frame 116 | | | |  |  |  |  |  |  |  |  |
| C10orf81 | NM_024889 | | NS | 3.9 | chromosome 10 open reading frame 81 | | | |  |  |  |  |  |  |  |  |
| C6orf15 | NM_014070 | | 4.1 | NS | chromosome 6 open reading frame 15 | | | |  |  |  |  |  |  |  |  |
| CNN3 | NM_001839 | | 12.0 | 2.1 | calponin 3, acidic | |  |  |  |  |  |  |  |  |  |  |
| DBP | U79283 | | 5.9 | 1.5 | D site of albumin promoter (D-box) binding protein | | | | | |  |  |  |  |  |  |
| DOC1 | NM_014890 | | 4.2 | NS | downregulated in ovarian cancer 1 | | | |  |  |  |  |  |  |  |  |
| DOCK9 | AL576253 | | 3.3 | 3.8 | dedicator of cytokinesis 9 | | |  |  |  |  |  |  |  |  |  |
| EHD2 | AI417917 | | 3.4 | 2.3 | EH-domain containing 2 | | |  |  |  |  |  |  |  |  |  |
| FLJ11259 | NM_018370 | | 2.5 | 14.4 | hypothetical protein FLJ11259 | | |  |  |  |  |  |  |  |  |  |
| PER3 | NM_016831 | | 3.4 | 2.7 | period homolog 3 (Drosophila) | | |  |  |  |  |  |  |  |  |  |
| RALGPS1 | NM_014636 | | 3.0 | NS | Ral GEF with PH domain and SH3 binding motif 1 | | | | |  |  |  |  |  |  |  |
| SACS | AI932370 | | NS | 4.1 | spastic ataxia of Charlevoix-Saguenay (sacsin) | | | | |  |  |  |  |  |  |  |
| SCEL | NM_003843 | | NS | 5.7 | sciellin |  |  |  |  |  |  |  |  |  |  |  |
| SHANK2 | BF435773 | | 4.8 | 2.1 | SH3 and multiple ankyrin repeat domains 2 | | | |  |  |  |  |  |  |  |  |
| TMEPAI | NM_020182 | | 4.9 | 2.6 | transmembrane, prostate androgen induced RNA | | | | |  |  |  |  |  |  |  |
| TRIM22 | AA083478 | | NS | 5.2 | tripartite motif-containing 22 | | |  |  |  |  |  |  |  |  |  |
| TXNIP | NM_006472 | | 3.4 | NS | thioredoxin interacting protein | | |  |  |  |  |  |  |  |  |  |
| UPK3B | NM_030570 | | NS | 5.8 | uroplakin 3B | |  |  |  |  |  |  |  |  |  |  |
| WFDC2 | NM_006103 | | NS | 3.2 | WAP four-disulfide core domain 2 | | | |  |  |  |  |  |  |  |  |
| PP1665 | AL041124 | | 6.4 | NS | hypothetical protein PP1665 | | |  |  |  |  |  |  |  |  |  |
|  |  | |  |  |  |  |  |  |  |  |  |  |  |  |  |  |
|  |  | |  |  |  |  |  |  |  |  |  |  |  |  |  |  |
|  |  | |  |  |  |  |  |  |  |  |  |  |  |  |  |  |
